# Surface hydroxide promotes CO_2_ electrolysis to ethylene in acidic conditions

**DOI:** 10.1038/s41467-023-37898-8

**Published:** 2023-04-25

**Authors:** Yufei Cao, Zhu Chen, Peihao Li, Adnan Ozden, Pengfei Ou, Weiyan Ni, Jehad Abed, Erfan Shirzadi, Jinqiang Zhang, David Sinton, Jun Ge, Edward H. Sargent

**Affiliations:** 1 Department of Electrical and Computer Engineering, University of Toronto, Toronto, ON M5S 3G4 Canada; 2 Key Lab for Industrial Biocatalysis, Ministry of Education, Department of Chemical Engineering, Tsinghua University, 100084 Beijing, China; 3 Department of Mechanical and Industrial Engineering, University of Toronto, Toronto, ON M5S 3G8 Canada; 4 Institute of Biomedical Health Technology and Engineering, Shenzhen Bay Laboratory, 518107 Shenzhen, China

**Keywords:** Electrocatalysis, Electrocatalysis, Materials for energy and catalysis

## Abstract

Performing CO_2_ reduction in acidic conditions enables high single-pass CO_2_ conversion efficiency. However, a faster kinetics of the hydrogen evolution reaction compared to CO_2_ reduction limits the selectivity toward multicarbon products. Prior studies have shown that adsorbed hydroxide on the Cu surface promotes CO_2_ reduction in neutral and alkaline conditions. We posited that limited adsorbed hydroxide species in acidic CO_2_ reduction could contribute to a low selectivity to multicarbon products. Here we report an electrodeposited Cu catalyst that suppresses hydrogen formation and promotes selective CO_2_ reduction in acidic conditions. Using in situ time-resolved Raman spectroscopy, we show that a high concentration of CO and OH on the catalyst surface promotes C-C coupling, a finding that we correlate with evidence of increased CO residence time. The optimized electrodeposited Cu catalyst achieves a 60% faradaic efficiency for ethylene and 90% for multicarbon products. When deployed in a slim flow cell, the catalyst attains a 20% energy efficiency to ethylene, and 30% to multicarbon products.

## Abstract (web summary)

Performing CO_2_ reduction in acidic conditions enables high CO_2_ utilization. Here, the authors report an electrodeposited Cu catalyst which achieves a 60% faradaic efficiency for ethylene and 90% for multicarbon products–both records for acidic CO_2_ reduction.

## Introduction

The electrocatalysis community has demonstrated high selectivity toward multicarbon (C_2+_) products in the CO_2_ reduction (CO_2_R) reaction^1–5^. In particular, the faradaic efficiency (FE) to ethylene (C_2_H_4_) in CO_2_R has increased at an impressive rate in systems operating in neutral and alkaline conditions^2,6–11^. Unfortunately, the single-pass carbon efficiency (SPCE) (i.e. the utilization of CO_2_) has till now remained low^12,13^. A primary cause is the rapid conversion of CO_2_ to (bi)carbonate in these mid- to high-pH conditions. This imposes a substantial energy cost penalty for carbonate regeneration and CO_2_ (re)capture^12,14^.

Performing CO_2_R in acidic conditions provides a potential route to high SPCE of CO_2_^12,14,15^. However, to date, the selectivity of CO_2_R in acidic conditions has been limited by the competing hydrogen evolution reaction (HER)^12,14^, which benefits from a faster kinetics and a lower reaction overpotential^16^. Consequently, the energy efficiency (EE) for producing C_2+_ chemicals in acidic CO_2_R has remained well below that in neutral and alkaline conditions.

The activity and selectivity of CO_2_R can be tuned by tailoring the interaction between reaction intermediates and the Cu surface^12,17–22^. Achieving a high coverage and long residence time of CO on the surface of the catalyst can help acidic CO_2_R compete successfully against HER^22^. Previous CO_2_R studies in neutral/alkaline conditions showed that adsorbed hydroxyl groups (OH^*^, * indicating surface site) form on the Cu surface during CO_2_R due to an increase in local alkalinity^23–25^. Such species have been shown to affect the adsorption energy of CO and the reaction rate of C–C coupling^26^. In acidic CO_2_R, the local pH at the electrode surface is near-neutral under moderate current densities^14^, a fact that will limit the concentration of surface OH^*^ and lead to low C_2+_ selectivity in acidic CO_2_R.

Here we focus therefore on a strategy to increase the concentration of OH^*^ on the catalyst surface during acidic CO_2_R. We report that it suppresses HER and enhances C–C coupling. Using density functional theory (DFT) calculations, we found an increase in the CO adsorption energy and a decrease in the barrier for C–C coupling when a sub-monolayer coverage of OH^*^ exists on the Cu surface. By tailoring synthesis conditions, we synthesized catalysts that maintain a high concentration of co-adsorbed CO and OH during acidic CO_2_R. Using time-resolved in situ Raman spectroscopy, we find that the residence time of CO on the catalyst surface increases in the presence of OH, a finding we correlate with higher C_2+_ selectivity and suppressed HER activity. The method of catalyst synthesis involves electrodepositing a Cu catalyst in situ during CO_2_ reduction (EC–Cu). This leads to achieving >50% and 90% FE for C_2_H_4_ and C_2+_, respectively, with 10 h of operating stability. When we add poly(amino acid) promotors, the catalyst achieves a C_2_H_4_ FE of 60% and a C_2_H_4_ EE of 20%.

## Results and discussion

### DFT calculations

**Fig. 1:**
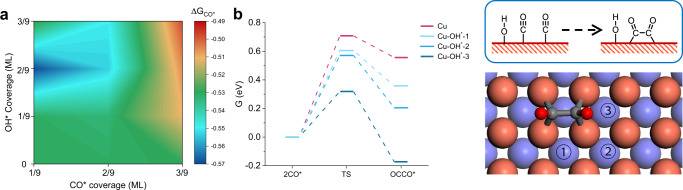
DFT calculations. **a** CO adsorption energy on Cu(100) surface with different OH and CO coverage. **b** Free energy profiles of CO dimerization on Cu(100) with and without adsorbed OH. Different positions of OH^*^ denoted as Cu-OH^*^-1, Cu-OH^−^-2, and Cu-OH^*^-3 were considered. The bottom right panel illustrates the OH^*^ positions in DFT models.

We first applied DFT calculations to investigate the free energy of CO adsorption (ΔG_CO*_) on Cu(100) as a function of the surface coverage of CO (θ_CO_) and OH (θ_OH_). We chose Cu(100) as the model surface due to its high activity for the C–C coupling reactions^10,27–29^ and investigated the dependence of ΔG_CO*_ on the θ_CO_ and θ_OH_ up to 3/9 of a monolayer (ML) (Supplementary Fig. S1). We found that ΔG_CO*_ is similar at different θ_CO_ without co-adsorbed OH (Fig. 1a). However, an increase in CO binding strength is observed for θ_CO_ between 1/9 and 2/9 ML when the θ_OH_ is at 2/9 ML, indicating that the ΔG_CO*_ can be fine-tuned with co-adsorbed OH. Comparing the barrier for C–C coupling with and without OH^*^ (Fig. 1b, Supplementary Figs. S2–5), we found a lower activation energy for CO dimerization and a lower free energy for OCCO (ΔG_OCCO*_) on Cu(100) with nearby adsorbed OH. A similar trend on the influence of OH^*^ in CO dimerization is also observed for Cu(111) (Supplementary Figs. S6−10). DFT calculation results suggest that introducing OH^*^ species in acidic CO_2_R could be a promising strategy to suppress HER and promote C_2+_ formation.

### In situ electrodeposition of catalysts during acidic CO_2_R

**Fig. 2:**
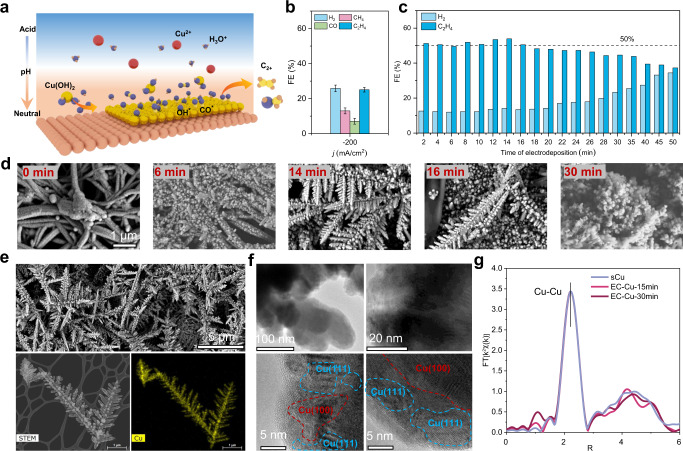
In situ electrodeposition in acidic CO2R. **a** Schematic of Cu(OH)_x_ mediated in situ electrodeposition during acidic CO_2_R in a flow cell. **b** Gas-phase product distribution of sCu in acidic CO_2_R. The error bars correspond to the standard deviation of three independent measurements. **c** FE_H2_ and FE_C2H4_ of in situ formed EC–Cu at different time of electrodeposition. The CO_2_R was performed at 200 mA cm^−2^ in 0.05 M H_2_SO_4_ and 2.5 M KCl containing the copper precursor (5 mM). The FE of CO and CH_4_ were less than 1% and not listed. **d** Morphology evolution of EC–Cu. Scanning electron microscope (SEM) image of a series of EC–Cu catalysts at different times of electrodeposition. **e** SEM and transmission electron microscopy (TEM) images and corresponding EDX mapping of Cu. **f** High-resolution TEM. **g** Fourier-transformed operando XAS spectra of the formations of EC–Cu with respect to time at 200 mA cm^−2^ in 0.05 M H_2_SO4 and 2.5 M KCl containing the copper precursor (3 mM).

Previous work reported that Cu catalysts with a surface OH^*^ are obtained at high pH by electrochemically reducing a pre-deposited Cu(OH)_2_ film^26^. We electrodeposited Cu catalysts (EC–Cu) in acidic conditions by reducing Cu^2+^ species during CO_2_R, which generates a locally alkaline condition (H_3_O^+^ mass transport limitation) necessary for the formation of Cu(OH)_x_ pre-catalyst and provides a reductive potential needed to reduce the as-formed Cu(OH)_x_ into Cu (Fig. 2a). The local formation of Cu(OH)_x_ pre-catalyst was found to be necessary to obtain high performance of EC–Cu catalysts—an observation arrived at by comparing with control samples prepared under various deposition conditions (Supplementary Fig. S11). The EC–Cu catalyst produced by Cu(OH)_*x*_–mediated in situ deposition differs in morphology and structure from the electrodeposited Cu catalysts obtained at locally acidic conditions. It also demonstrates a higher C_2+_ selectivity (vide infra).

We performed electrodeposition of Cu on a sputtered Cu (sCu) covered polytetrafluoroethylene (PTFE) gas diffusion layer (sCu/PTFE) in a flow cell during acidic CO_2_R (Fig. 2a). The sCu catalyst, a control, showed a C_2_H_4_ FE of 25% and a H_2_ FE of 26% (Fig. 1b)^9,11^. During electrodeposition, the selectivity of EC–Cu towards C_2_H_4_ improved, reaching ~50% C_2_H_4_ FE, while the HER selectivity was reduced substantially (Fig. 2c). The FE to C_1_ products (CO and CH_4_) was <1%, lower than that of sCu. We also observed that deposition duration of >14 min has a negative effect on the FE of C_2_H_4_ with a concomitant increase in HER.

We relate the time-dependent CO_2_R activity to the morphology change of the deposited catalysts during synthesis. Scanning electron microscopy (SEM) images of EC–Cu show that dendritic structures gradually form on the sCu/PTFE surface with a longer deposition duration (Fig. 2d, e). A complete dendritic structure was formed at 14 min, corresponding to the best performance.

In a Cu^2+^ free electrolyte, the optimally deposited EC–Cu catalyst (at 14 min) maintained high CO_2_R selectivities, excluding CO_2_R catalysis by solvated Cu^2+^ ions (Supplementary Fig. S12). Transmission electron microscopy (TEM) shows that different Cu facets exist (Fig. 2f). This is consistent with OH^−^ adsorption experiments showing a mixture of (100), (110), and (111) surface crystal orientations on EC–Cu, and the ratios of these facets differ greatly from that of sCu (Supplementary Fig. S13). Operando X-ray absorption spectroscopy (XAS) was used to track the oxidation state and local bonding configuration of Cu as a function of deposition duration (Fig. 2g, Supplementary Fig. S14). The EC–Cu catalysts were metallic and showed Cu coordination similar to that of sCu.

### Experimental mechanistic studies

**Fig. 3:**
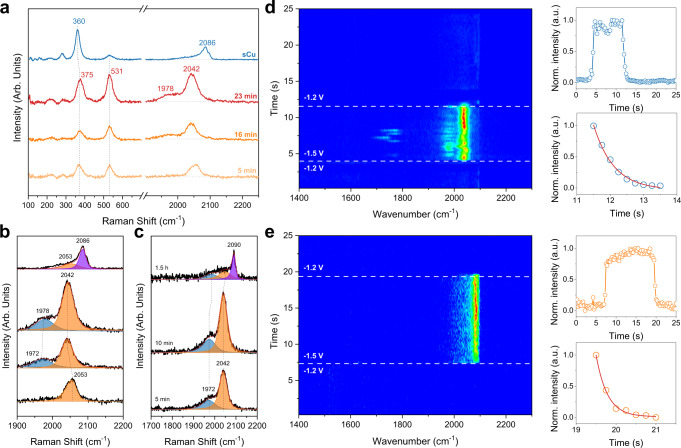
In situ Raman spectroscopy of sCu and EC–Cu catalysts. **a** Raman spectra of sCu and EC–Cu after different electrodeposition time. **b** Deconvolved spectra of the ν(CO_atop_) region of (**a**). **c** Deconvolved spectra of the ν(CO_atop_) region for EC–Cu catalyst after 5, 10, and 90 min of CO_2_ electrolysis. **d** Time-resolved in situ Raman spectra of EC–Cu under −1.2 and −1.5 V applied potentials. The top right panel summarizes the normalized intensity of the 2042 cm^−1^ peak as a function of time. The bottom right panel illustrates the time-dependent change of the normalized intensity for the 2042 cm^−1^ peak after a potential step to −1.2 V. The red curve is an exponential fit to the intensity decay. **e** Time-resolved in situ Raman spectra of sCu under −1.2 and −1.5 V applied potentials. The top right panel plots the normalized intensity of the 2082 cm^−1^ peak as a function of time. The bottom right panel illustrates the time-dependent change of the normalized intensity for the 2082 cm^−1^ peak after a potential step to −1.2 V. The red curve is an exponential fit to the intensity decay. In situ Raman spectra were collected using a 785 nm laser. The time-resolved spectra were collected every 250 ms.

We employed time-resolved in situ Raman spectroscopy to probe the surface-adsorbed CO—an important reaction intermediate in CO_2_R. When CO chemisorbs on the Cu catalysts, its characteristic stretching frequency (ν(CO_atop_)) ranges from 1850−2100 cm^−1^. The values of ν(CO_atop_) depend on multiple factors, including the adsorption site (atop vs. bridge sites) and surface CO coverage (θ_CO_). Based on the peak positions, we assign the peaks at 2082 and 2042 cm^−1^ to CO molecules adsorbed at the atop sites of the Cu surface on sCu and EC–Cu, respectively (Fig. 3a). The high-frequency band (HFB) at 2082 cm^−1^ is assigned to CO_atop_ on isolated step sites, and the lower frequency band (LFB) at 2042 cm^−1^ is attributed to CO_atop_ on terrace sites^17^ (Fig. 3b, Supplementary Fig. S15). The lower ν(CO_atop_) frequency on EC–Cu indicates stronger CO_atop_ binding on its surface than sCu. This result is consistent with a higher ν(Cu–CO) frequency observed for EC–Cu at 375 cm^−1^ compared to sCu at 360 cm^−1^ (see Supplementary Note 1 for additional discussion)^30^. A consequence of stronger CO_atop_ binding on EC–Cu is a longer residence time on the catalyst surface, which could benefit CO–CO coupling for C_2+_ product formation. We evaluate CO residence time on the Cu surface by monitoring the decrease in ν(CO_atop_) peak intensity after changing the applied potential from high (−1.5 V) to low (−1.2 V) rate conditions. For sCu and EC–Cu, the ν(CO_atop_) peak intensity decays exponentially with time constants (τ) of 0.33 and 0.61 s, respectively (Fig. 3d, e). Similar τ values are also observed for the corresponding Cu–CO peak at 360 and 378 cm^−1^ (Supplementary Fig. S16, S17). The larger τ values of the 2042 and 378 cm^−1^ peaks indicate a longer residence time of CO_atop_ on the surface of EC–Cu, which can promote C–C coupling in addition to having a high θ_CO_. This effect is also in line with the higher C_2+_ FE for the EC–Cu (>90%) compared with sCu (<50%). Furthermore, the degraded EC–Cu samples (<65% C_2+_ FE) show a high-frequency ν(CO_atop_) band with short τ, reaffirming the importance of longer residence time for adsorbed CO_atop_ (Fig. 3c, Supplementary Figs. S18, 19).

The larger τ value for EC–Cu is closely associated with the surface OH^*^ group. The greater intensity of the Cu-OH peak at 531 cm^−1^ for EC–Cu suggests a greater concentration of OH^*^ at the surface compared to sCu under the same applied potential (Fig. 3a). DFT calculations show that the ΔG_CO*_ can increase with the number of nearby adsorbed OH. Experimentally, we observed the interactions between co-adsorbed CO and OH based on a red shift in the ν(CO_atop_) peak position to 2042 cm^−1^ and a lower frequency shoulder at 1972 cm^−1^ (Fig. 3a, Supplementary Note 1)^26^. Combining in situ characterization of surface OH and CO_atop_, we propose that EC–Cu enables high surface coverage of co-adsorbed OH and CO, which increases the residence time of CO on the catalyst surface and promotes C–C coupling. Evidence from Raman spectroscopy indicates the presence of OH^*^ on the surface of Cu catalysts. However, since we cannot observe and quantify the concentration of locally generated hydroxyl ions by Raman spectroscopy, we do not rule out the effect of this species on the CO_2_R selectivity (Supplementary Figs. S20, 21).

### Acidic CO_2_R performance

**Fig. 4:**
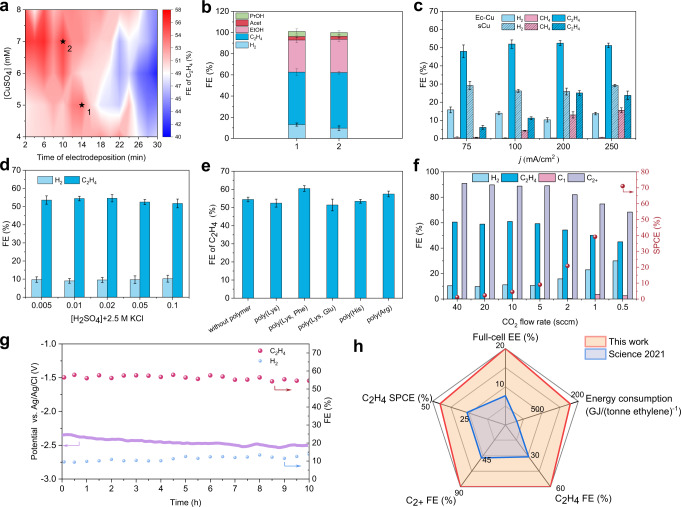
CO2R performance of EC–Cu catalysts. **a** FE_C2H4_ of EC–Cu obtained from different electrodeposition durations and Cu precursor concentrations. **b** Acidic CO_2_R product distribution of EC–Cu−1 (5 mM CuSO_4_, 14 min) and EC–Cu-2 (7 mM CuSO_4_, 10 min) in (**a**). **c** The gas-phase product distribution of EC–Cu (7 mM CuSO_4_, 10 min) and sCu in acidic CO_2_R at different current densities. **d** FE_H2_ and FE_C2H4_ of EC–Cu-2 when using different concentrations of H_2_SO_4_ in the catholyte. **e** Performance of different poly(amino acid)s modified EC–Cu-2. **f** SPC of acidic CO_2_R. **g** Cell voltage, FE_C2H4_ and FE_H2_ of acidic CO_2_R in a flow cell. **h** Full-cell EE, energy consumption, C_2_H_4_ FE, C_2+_ FE and C_2_H_4_ SPCE compared to the best prior results for acidic CO_2_R. All the error bars correspond to the standard deviation of three independent measurements.

We prepared EC–Cu on different conductive substrates and found that sCu/PTFE resulted in the highest FE of C_2_H_4_ and C_2+_. The interaction between the 200 nm Cu film (sCu) and electrodeposited Cu species (EC–Cu) has little influence on the performance of the catalysts (Supplementary Fig. S22). The thickness of Cu in sCu also has a minimal impact on the FE of C_2_H_4_, but it significantly affects the stability of EC–Cu. Additionally, the substrates’ structure and composition can influence EC–Cu performance (Supplementary Fig. S23)^31–33^. A 200 nm Cu film deposited on a PTFE-coated gas diffusion layer was used as the substrate in all experiments unless otherwise specified. We found that the optimal deposition time of EC–Cu decreased with greater CuSO_4_ concentrations (Fig. 4a). We identified two deposition conditions that produce highly selective catalysts for C_2_H_4_ as EC–Cu-1 and EC–Cu-2 (optimal electrodeposition duration at 5 mM and 7 mM CuSO_4_). The EC–Cu catalysts synthesized using the second deposition condition achieved a maximum C_2_H_4_ FE of 56%. Over a long-time operation, the average FE for C_2_H_4_, C_2+,_ and H_2_ was maintained at 53%, 90%, and 10%, respectively (Fig. 4b).

In previous acidic CO_2_R works, high current densities were necessary to increase the local pH and promote C–C coupling on Cu catalysts, and a high K^+^ concentration was employed to suppress HER (Supplementary Fig. S24)^12,14,34^. This was observed for sCu, which showed a lower FE to C_2_H_4_ at lower current densities (Fig. 4c, <10% at 75 mA/cm^2^). In contrast, EC–Cu demonstrated >45% FE to C_2_H_4_ from 75 to 250 mA/cm^2^ and was able to achieve high C_2_H_4_ FE at lower K^+^ concentrations (Fig. 4c, d and Supplementary Figs. S25, 26). We attribute this high selectivity partially to a high surface OH^*^ concentration during CO_2_R, which stabilizes CO and reduces the C–C coupling barrier.

The stability of EC–Cu was also improved compared with sCu, from less than 0.5 h to 10 h (Supplementary Figs. S27–29). We note that the degradation of dendritic structures in EC–Cu accompanies a performance decrease, which correlates with a decrease in surface OH, and a shorter CO residence time (Supplementary Figs. S18, 19). To further increase the FE of C_2_H_4_, we introduced amide-bearing polymers during the catalyst deposition process. We evaluated five poly(amino acids) containing abundant amide groups and different functional side chains and found that poly(Lys, Phe) showed a stable C_2_H_4_ FE of 60% (Fig. 4e, Supplementary Figs. S30, 31). A similar promoting effect of amine/amide functional groups toward the C_2_H_4_ selectivity in CO_2_R has been reported previously^6,35^. Deploying the poly(Lys, Phe)-modified EC–Cu catalyst, we obtained a SPCE of 70% towards C_2+_ products (Fig. 4f) and stable operation for 10 h with 55% FE of C_2_H_4_ in acidic conditions (Fig. 4g). Based on a full cell voltage of 3.4 V (Supplementary Fig. S32), the EE towards C_2_H_4_ is 20%, which is 1.6-fold greater than the previous acidic CO_2_-to-C_2_H_4_ record. Figure 3h summarizes the result in comparison with the previous best acidic CO_2_R performance (Supplementary Table 1–3). Based on the performance summary in Fig. 3h, we achieved CO_2_-to-C_2_H_4_ conversion with an energy requirement of ~266 GJ ton^−1^, lower than previous neutral and alkaline CO_2_R systems^13^. Despite the promising performance, practical implementation requires further improvements in stability and selectivity.

In summary, we report an EC–Cu catalyst that selects for C_2_H_4_ and C_2+_ products in acidic CO_2_R. Preparation via in situ electrodeposition led to a higher surface coverage of OH, and we found that this species interacts with the CO intermediate and increases the CO binding energy. This observation is consistent with DFT calculations showing that sub-monolayer coverage of OH can increase the free energy of CO adsorption and reduce the barrier for C–C coupling. Implementing the EC–Cu catalyst in acidic CO_2_R achieves a high FE toward multicarbon products (60% for C_2_H_4_, 90% for C_2+_), a high SPCE of CO_2_ to C_2+_ products (70%), and a high EE (20% for C_2_H_4_, 30% for C_2+_) simultaneously.

## Methods

### Materials

A high-purity Cu target (99.99%, Kurt J. Lesker Company, EJTCUXX403A2) was used in the fabrication of sputtered Cu catalysts. Sulfuric acid (H_2_SO_4_, Fischer Chemicals, A300S), copper(II) sulfate pentahydrate (CuSO_4_ · 5H_2_O, Aldrich, 209198), and potassium chloride (KCl, Aldrich, P3911) were used in the preparation of supporting electrolytes. Polytetrafluoroethylene (PTFE) gas diffusion layers (GDL) with 450 nm pore size were purchased from Beijing Zhongxingweiye Instrument Co., Ltd. Nafion 117 membrane was purchased from the FuelCell Store. All poly(amino acid)s: Poly(l-Lys, l-Phe) 1:1 hydrobromide (P3150), Poly(l-Glu, l-Lys) 1:4 hydrobromide (P8619), Poly(l-His) (P9386), Poly(l-Lys) hydrobromide (P2636), Poly(l-Arg) hydrochloride (P4663) were purchased from Sigma Aldrich.

### In situ electrodeposition

Magnetron sputtering (Angstron Engineering, Nextdep) was used to deposit 200 nm or 500 nm of Cu catalyst on a PTFE GDL at a rate of 1 Å/s in a high vacuum condition. EC–Cu samples were prepared by electrodeposition under acidic CO_2_R conditions. Catalysts were electrodeposited at a constant current density of −200 mA cm^-2^ for a certain duration on the sputtered Cu in a three-electrode flow cell. The catholyte consisted of 3~8 mM CuSO_4_, 0.05 M H_2_SO_4_, and 2.5 M KCl. 0.05 M H_2_SO_4_ was used as the anolyte. For electrodeposition containing poly(amino acid), each polymer was added to the catholyte at 0.1 mg/ml concentration. The catholyte and anolyte chambers were separated by a cation exchange membrane (CEM, Nafion 117®). An Ag/AgCl reference electrode (3 M KCl) and a platinum mesh (Fisher Scientific, AA41814FF) counter electrode were used. The in situ nature of the catalyst deposition allows us to monitor the performance evolution of the formed catalysts in real-time. In situ electrodeposition was performed under the same electrochemical conditions as acidic CO_2_R except for the addition of Cu precursor (CuSO_4_) to the catholyte. After the deposition time interval, a Cu^2+^-free catholyte (0.05 M H_2_SO_4_, 2.5 M KCl) was introduced to the reactor, which terminated the electrodeposition process, allowing continued evaluation of the in situ formed catalysts.

### Catalyst characterization

Scanning electron microscopy (SEM, Hitachi S3500) and transmission electron microscopy (TEM, Hitachi HF3300) were used to characterize the crystallinity and morphology of the in situ formed Cu catalysts. The XAS measurements were performed with a modified flow cell in the Soft X-ray Micro-Characterization Beamline (SXRMB) at the Canadian Light Source (Saskatoon, Canada), which is equipped with a water-cooled Si (111) and InSn (111) double-crystal monochromator covering a photon energy range from 1.7 to 10.0 keV. The XAS data was processed with Demeter (v.0.9.26)^36^. In situ Raman spectroscopy (Renishaw Invia Raman) was performed to investigate CO speciation on sCu and EC–Cu during CO_2_R.

### CO_2_ reduction in a flow cell

Three chambers constitute the flow cell setup: anolyte chamber, catholyte chamber, and gas flow chamber. Each compartment has an inlet and outlet. The electrolytes were circulated through the flow cell using peristaltic pumps. CO_2_ (Linde Gas) flowing through the gas flow chamber was controlled using a mass flow controller (Brooks Instrument, GF100). The CO_2_ flow rate was fixed at 40 standard cubic centimeters per minute (sccm) unless stated otherwise. During the CO_2_R reaction, the catalyst-coated working electrodes, Nafion 117 membrane, and a platinum mesh (Fisher Scientific, AA41814FF) counter electrode were sandwiched between the three chambers. The working electrode potential was measured against Ag/AgCl reference electrode (CH Instruments, CHI111P) that was inserted into the catholyte chamber.

### CO_2_ reduction in a full cell

A slim flow cell was used to measure the full cell performance. The slim flow cell also contains anolyte, catholyte, and gas flow compartments. The catholyte chamber is made of PEEK and has a total thickness of 1/16”. The reduced dimension is intended to lower ohmic losses between the cathode and the anode. In the full cell measurement, oxygen evolution reaction takes place on the anode and it was catalyzed by iridium oxide catalyst supported on titanium felt (IrO_*x*_–Ti)^37^. The IrO_*x*_–Ti anode electrodes were fabricated according to the following steps: (1) immersing the high porosity titanium fiber felts (Fuel Cell Store, porosity approximately 70-73%) into an ink of 2-propanol, iridium (IV) chloride dehydrate (Premion®, 99.99%, metal basis, Ir 73%, Alfa Aesar), and 0.1 M hydrochloric acid (ACS reagent; 37 vol%), (2) drying at 100 °C for 15 min and sintering at 500 °C for 15 min, and (3) repeating the first two steps until a final Ir mass loading of 1.5 mg/cm^2^ is achieved.

### Electrochemical measurement

All electrochemical measurements were performed using a potentiostat (Autolab PGSTAT302N). The CO_2_R performance was evaluated in a flow cell (three-electrode setup) or a slim flow cell (full cell, two-electrode setup) under galvanostatic modes. Gas-phase products were quantified using gas chromatography (GC, Shimadzu, GC-2014) equipped with a thermal conductivity detector (TCD) for the detection of H_2_, O_2_, N_2_, and CO, and a flame ionization detector (FID). Helium (Linde, 99.999%) was used as the carrier gas. Liquid products were analyzed using ^1^H NMR spectroscopy (600 MHz, Agilent DD2 NMR Spectrometer) with water suppression. Dimethyl sulfoxide (DMSO) was used as the internal reference and deuterium oxide (D_2_O) as the lock solvent. The Faradaic efficiency (FE) of the gas product was calculated based on the following equation:

1 $${{{{{{\rm{FE}}}}}}}=\frac{{nFvr}}{i{V}_{m}}$$

where *n* is the number of electrons transferred, *F* is the Faraday constant, *v* is the CO_2_ flow rate, *r* is the concentration of the gas product in parts-per-million (ppm), *i* is the total current and *V*_*m*_ is the unit molar volume of gas.

The energy efficiency (EE) for the formation of ethylene is calculated as follows:

2 $${{{{{{\rm{EE}}}}}}}=\frac{{{{{{{{\rm{FE}}}}}}}}_{{{{{{\rm{C}}}}}}_2{{{{{\rm{H}}}}}}_4}*({E}_{a}^{o}-{E}_{c}^{o})}{{V}_{{{{{{\rm{full}}}}}}}}$$

where FE_C2H4_ denotes the FE of ethylene (C_2_H_4_), *E*^o^_a_ and *E*^o^_c_ are the standard reduction potentials for the anode and cathode (CO_2_-to-ethylene) reactions, respectively.

The single-pass carbon efficiency (SPCE) of CO_2_ for a particular product is determined as follows:

3 $${{{{{{\rm{SPCE}}}}}}}=\frac{60s*\frac{j}{{nF}}}{v(L/\!\min )*1\left(\min \right)/24.05(\frac{L}{{{{{{{\rm{mol}}}}}}}})}$$

where *j* is the partial current density of a specific product, *n* is the number of electrons transferred for every molecule of the product

### In situ Raman spectroscopy

A custom-made cell was used to carry out in situ Raman spectroscopy. In an epi-illumination configuration, a 785 nm laser was used as the excitation source. The laser power was kept lower than 0.20 mW in all experiments to minimize sample damage. The scattered Raman light was collected by a water immersion objective (Leica, 63×, NA 0.9). Raman spectrometer calibration was done with a Si standard.

### Electrochemical OH^−^ adsorption

Electrochemical OH^−^ adsorption was performed in an N_2_-saturated 1 M KOH electrolyte. Linear sweep voltammetry was carried out between −0.2 and +0.6 V versus RHE with a sweep rate of 100 mV s^−1^. Before the experiment, all copper catalysts were reduced at −0.6 V versus RHE for 3 min.

### DFT Calculations

All density functional theory (DFT) calculations were carried out using the Vienna ab initio simulation package (VASP)^38–41^. The generalized gradient approximation was used with the Perdew–Burke–Ernzerhof exchange-correlation functional^42^. The projector-augmented wave method^43,44^ was used to describe the electron-ion interactions and the cut-of energy for the plane wave basis set was 450 eV. To illustrate the long-range dispersion interactions between the adsorbates and catalysts, the D3 correction method was employed^45,46^. Brillouin zone integration was accomplished using a 3 × 3 × 1 and 2 × 3 × 1 Monkhorst-Pack k-point mesh for CO adsorption energy calculations and C–C coupling calculations. A vacuum region of more than 15 Å thickness was included along the perpendicular direction to avoid artificial interactions.

A periodic six-layer model and p(3 × 3) or p(6 × 3) super cell were chosen. A monolayer of charged water molecules was included in C–C coupling calculations. Adsorption geometries of the different states were optimized by a force-based conjugate gradient algorithm, whereas the transition states were located using the climbing image-nudged elastic band method^47^. During the calculations, the three lower layers were fixed and the three upper layers together with water molecules and adsorbates were allowed to relax. The Gibbs free energy (∆G) was calculated by converting the electronic energy using the equation: ∆*G* = ∆*E* + ∆ZPE + ∫ ∆*C*_*p*_d*T* − *T∆S*, where ∆*E*, ∆ZPE, ∆*C*_*p*_, and ∆*S* are the differences in electronic energy, zero-point energy, heat capacity and entropy, respectively, and *T* was set to room temperature (298.15 K).

### Energy assessment for the acidic CO_2_R electrolyzer

Energy assessment of the acidic CO_2_R electrolyzer was performed using an energy evaluation model similar to that reported in refs. ^13,48^. This section provides an overview of the model used to obtain the energy intensity of ethylene production in an acidic CO_2_R electrolyzer. To calculate the energy intensity, we utilized the experimentally achieved performance metrics under various operating conditions (for example, under various CO_2_ input flow rates). The metrics inputted to the energy model include Faradaic efficiency, single-pass conversion efficiency, full-cell voltage, and current density—all towards ethylene (which is the major product of the acidic CO_2_R system). The model considers the presence of hydrogen (the product of competing HER) in addition to ethylene and CO_2_ at the cathode gas stream. The model also considers oxygen (the product of oxygen evolution) as the only product in the anode gas stream. The model considers a pressure swing adsorption (PSA) gas separation unit at the cathodic downstream to recover ethylene from the hydrogen side product and unreacted CO_2_. The CO_2_ recovered from the downstream of the cathode is considered to be returned to the cathode inlet for utilization in CO_2_R. An electrolyte requirement of 100 L per m^2^ of the active electrode geometric area is considered. This consideration is based on an estimation of the electrolyte requirement for a lab-scale electrolyzer with a geometric flow field area of 5 cm^2^. The electrolyte is considered to be circulating through a closed loop and used for 1 year without replacement. An example calculation for the energy intensity associated with the electrolyzer electricity and cathode separation is provided in Supplementary Note 2.

## Supplementary information

Supplementary Information

## Data Availability

All the data supporting the findings of this study are available within the article and its Supplementary Information. All other relevant source data are available from the corresponding authors upon reasonable request.
