# Supplementary material for: Surface hydroxide promotes CO2 electrolysis to ethylene in acidic conditions
Source: Nat Commun. 2023 Apr 25;14:2387. doi: 10.1038/s41467-023-37898-8 (PMC10130127; doi:10.1038/s41467-023-37898-8)
Supplement: Supplementary file 1 — Supplementary Information [file 41467_2023_37898_MOESM1_ESM.pdf]

Supplementary Information for

Surface Hydroxide Promotes CO_2 Electrolysis to Ethylene in Acidic Conditions

Yufei Cao[1,2†], Zhu Chen[1†], Peihao Li[1], Adnan Ozden[3], Pengfei Ou[1], Weiyan Ni[1], Jehad Abed[1], Erfan Shirzadi[1], Jinqiang Zhang[1], David Sinton[3], Jun Ge[2,4]*, Edward H. Sargent[1]*

[1]Department of Electrical and Computer Engineering, University of Toronto, Toronto, ON M5S 3G4, Canada

[2]Key Lab for Industrial Biocatalysis, Ministry of Education, Department of Chemical Engineering, Tsinghua University, Beijing 100084, China

[3]Department of Mechanical and Industrial Engineering, University of Toronto, Toronto, ON M5S 3G8, Canada

[4]Institute of Biomedical Health Technology and Engineering, Shenzhen Bay Laboratory, Shenzhen 518107, China

[†]These authors contributed equally

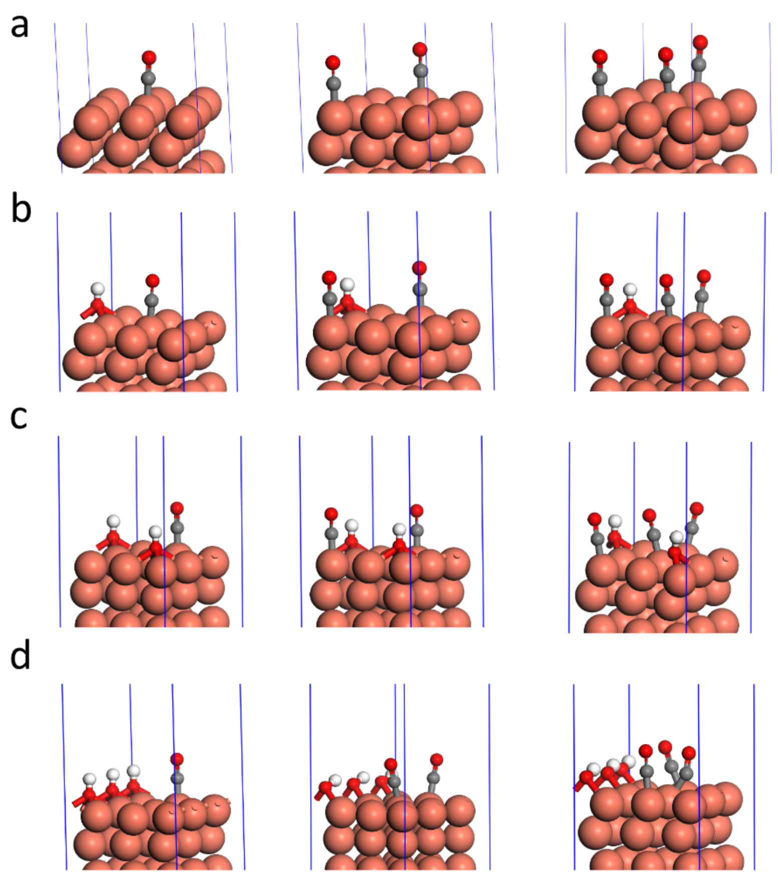

Supplementary Figure 1. DFT calculated conformation of co-adsorbed CO and OH. CO coverage increased from 1/9 to 3/9 ML. (a) Cu(100) without OH^*. (b) Cu(100) with one OH^*. (c) Cu(100) with two OH^*. (d) Cu(100) with three OH^*.

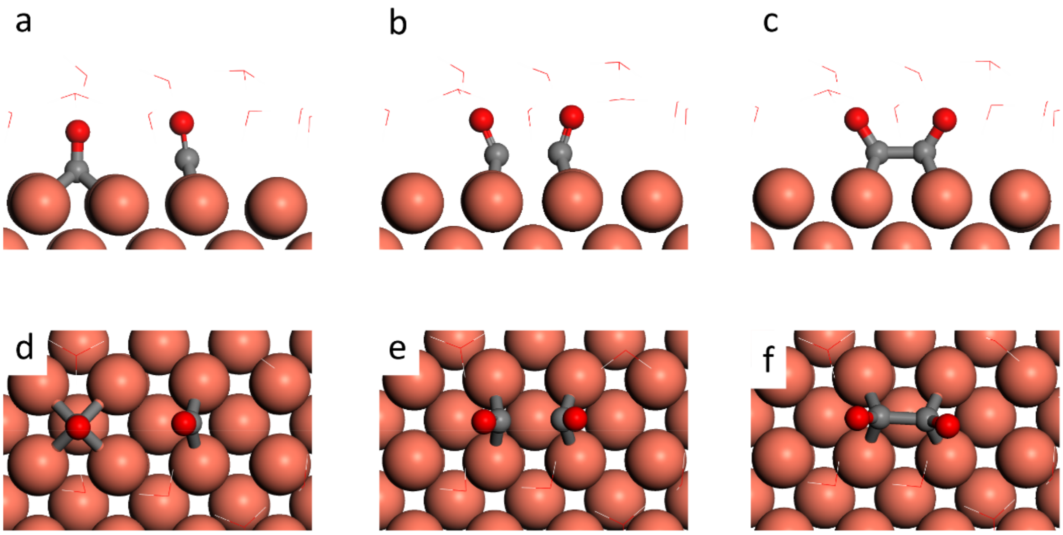

Supplementary Figure 2. Geometries of CO dimerization on Cu(100). Side views of initial state a, transition state b, final state c, and top views of initial state d, transition state e, final state f. Red, grey and orange balls stand for oxygen, carbon and copper atoms, respectively. Water molecules are shown as lines.

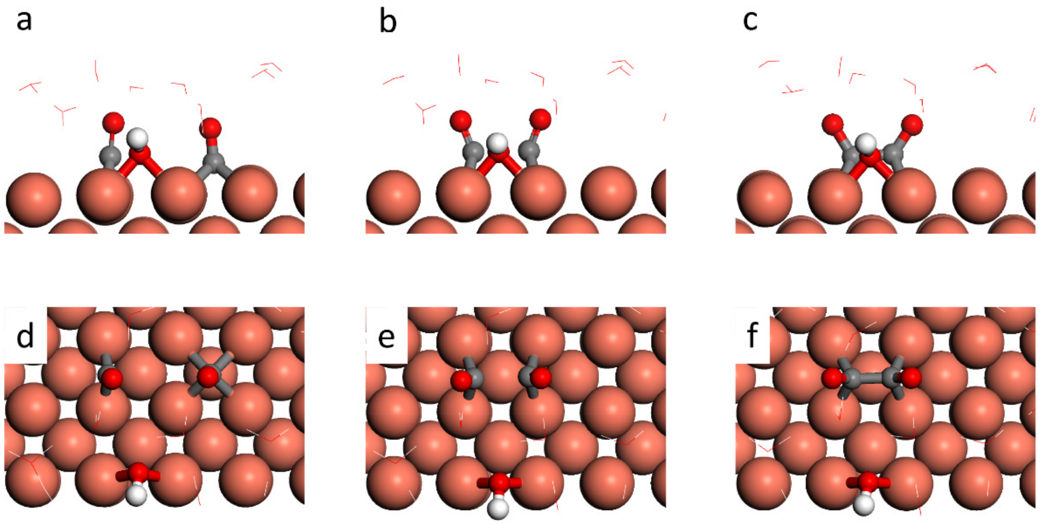

Supplementary Figure 3. Geometries of CO dimerization on Cu(100) with one surface OH^* at position 1. Side views of initial state a, transition state b, final state c, and top views of initial state d, transition state e, final state f.

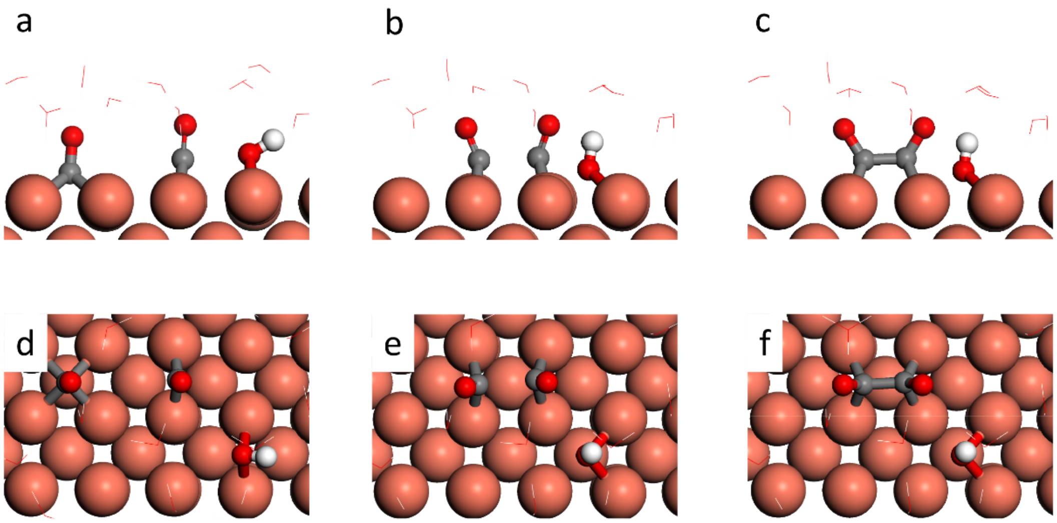

Supplementary Figure 4. Geometries of CO dimerization on Cu(100) with one surface OH^* at position 2. Side views of initial state a, transition state b, final state c, and top views of initial state d, transition state e, final state f.

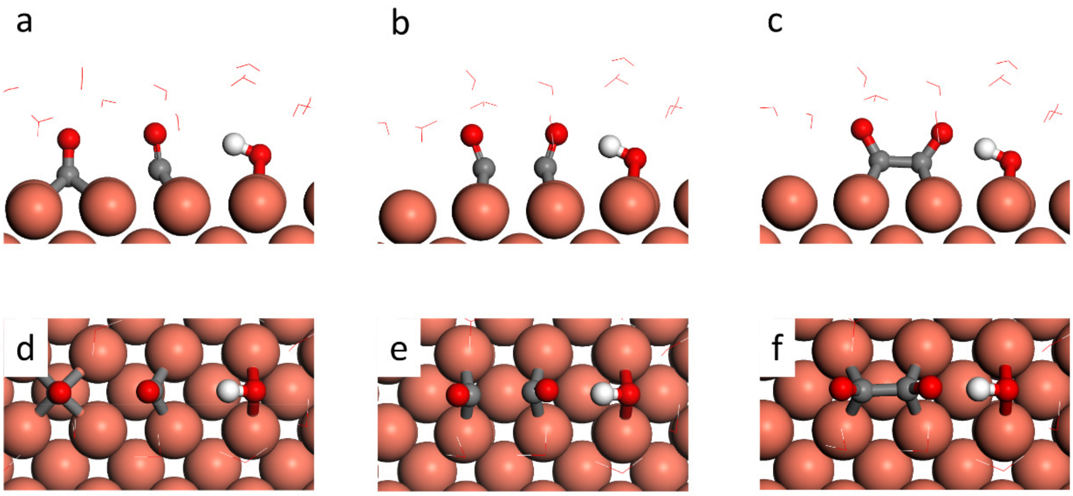

Supplementary Figure 5. Geometries of CO dimerization on Cu(100) with one surface OH^* at position 3. Side views of initial state a, transition state b, final state c, and top views of initial state d, transition state e, final state f.

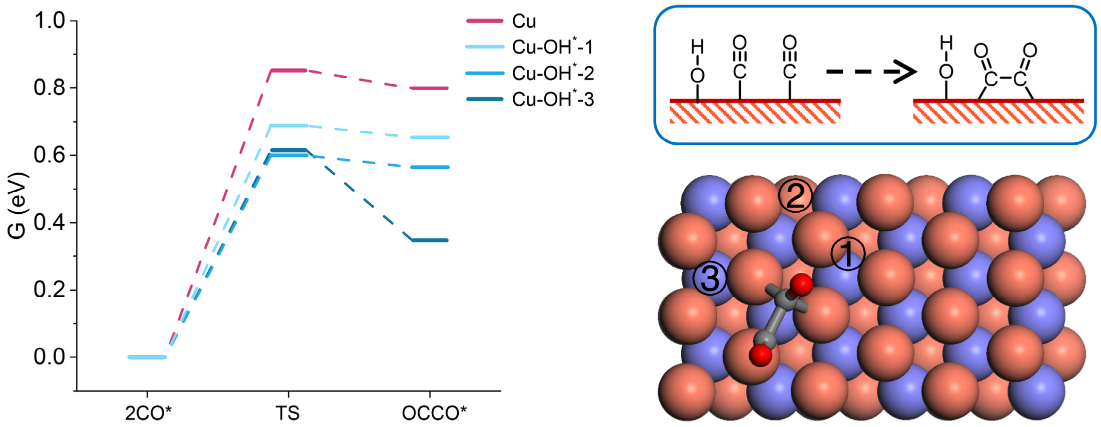

Supplementary Figure 6. Free energy profiles of CO dimerization on Cu(111) with and without adsorbed OH. Different positions of OH^* denoted as Cu-OH^*-1, Cu-OH^*-2, and Cu-OH^*-3 were considered. The bottom right panel illustrates the OH^* positions in DFT models.

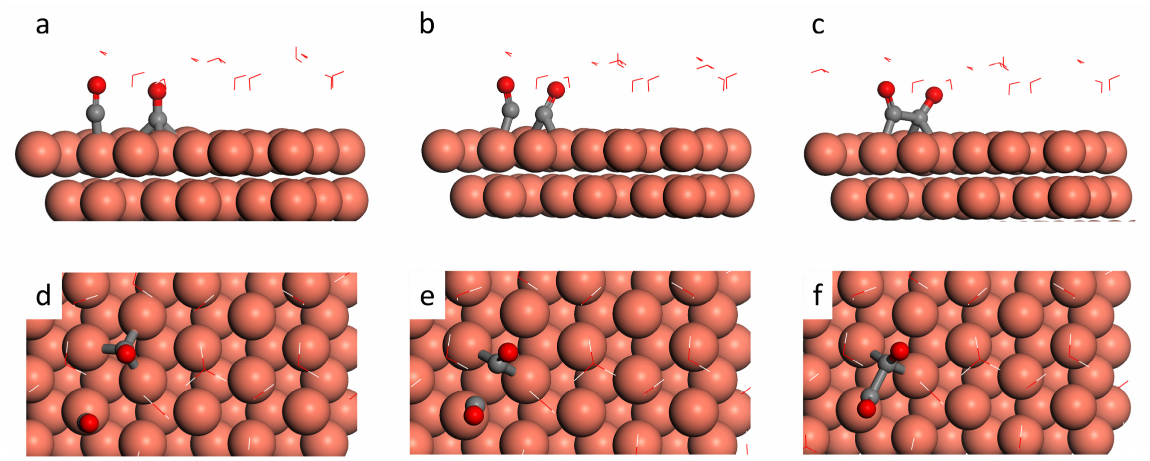

Supplementary Figure 7. Geometries of CO dimerization on Cu(111). Side views of initial state a, transition state b, final state c, and top views of initial state d, transition state e, final state f.

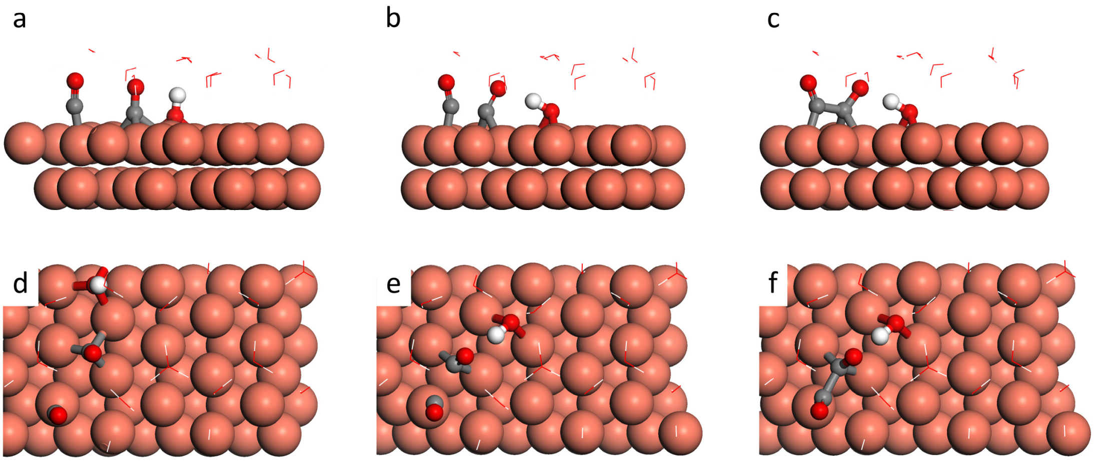

Supplementary Figure 8. Geometries of CO dimerization on Cu(111) with one surface OH* at position 1. Side views of initial state a, transition state b, final state c, and top views of initial state d, transition state e, final state f.

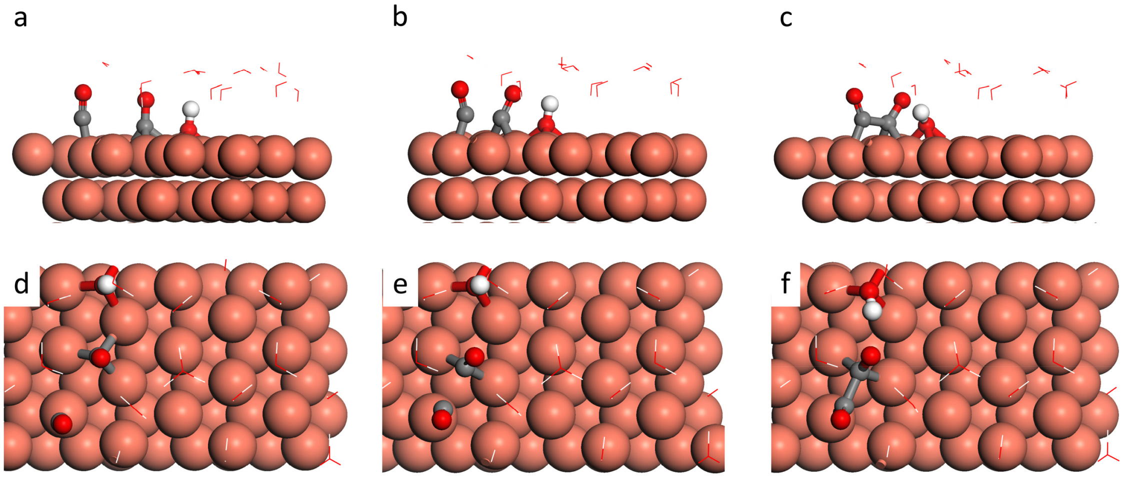

Supplementary Figure 9. Geometries of CO dimerization on Cu(111) with one surface OH* at position 2. Side views of initial state a, transition state b, final state c, and top views of initial state d, transition state e, final state f.

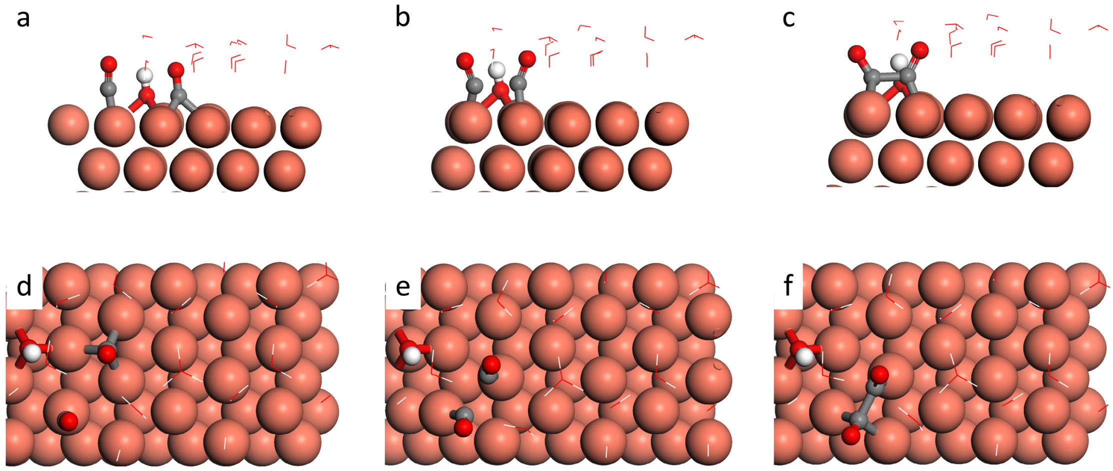

Supplementary Figure 10. Geometries of CO dimerization on Cu(111) with one surface OH* at position 3. Side views of initial state a, transition state b, final state c, and top views of initial state d, transition state e, final state f.

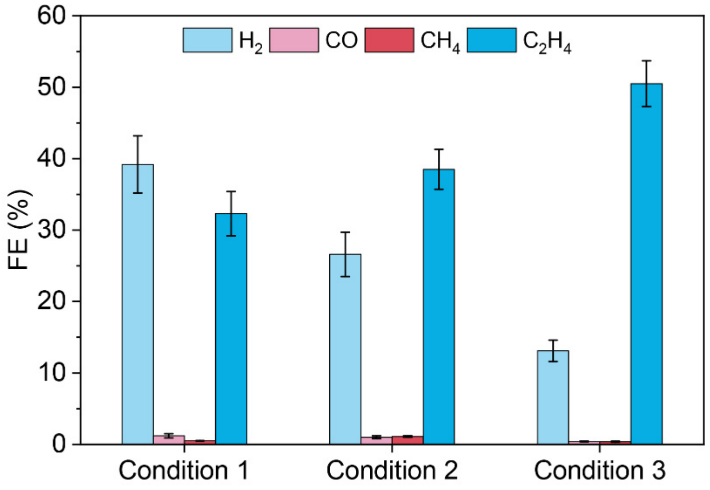

Supplementary Figure 11. Control samples prepared under various deposition conditions. Condition 1: catholyte 0.05 M H_2SO_4 + 5 mM $CuSO_4$, anolyte 0.05 M H_2SO_4, electrodeposition for 5 min. Condition 2: catholyte 1 M H_3PO_4 + 2.5 M KCl + 5 mM $CuSO_4$, anolyte 0.05 M H_2SO_4, electrodeposition for 14 min. Condition 3: catholyte 0.05 M H_2SO_4 + 2.5 M KCl + 5 mM $CuSO_4$, anolyte 0.05 M H_2SO_4, electrodeposition for 14 min. After electrodeposition, the obtained catalysts were tested in acidic CO_2R (catholyte: 0.05 M H2SO4 + 2.5 M KCl, anolyte: 0.05 M H_2SO_4). The error bars correspond to the standard deviation of three independent measurements.

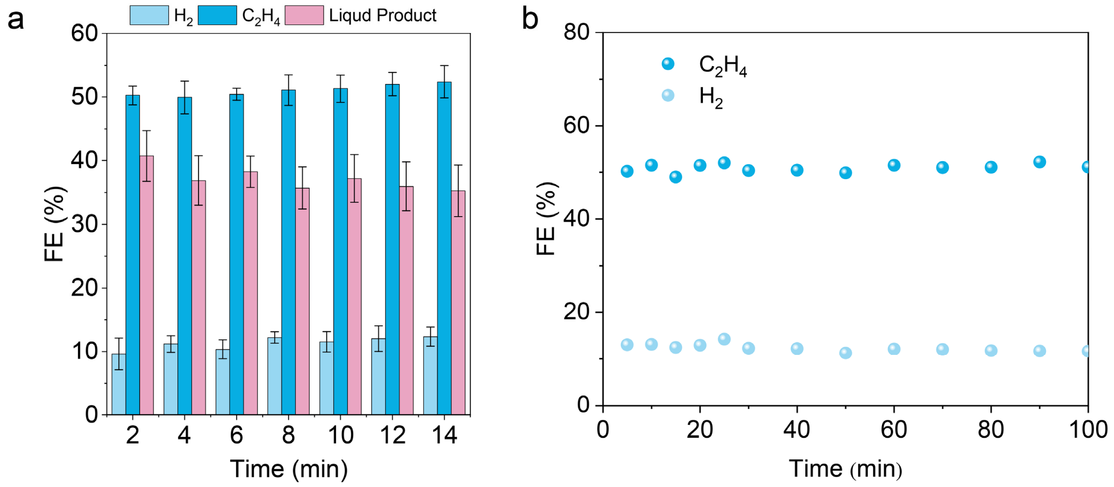

Supplementary Figure 12. *In situ* electrodeposition in acidic CO_2R. (a) Performance of the *in situ* formed catalysts during 14 min-long electrodeposition. The catholyte used was 0.05 M H_2SO_4 + 2.5 M KCl + 5 mM $CuSO_4$, and the anolyte was 0.05 M H_2SO_4. The CO_2 flow rate was 40 sccm. The error bars correspond to the standard deviation of three independent measurements. (b) After 14 min-long electrodeposition, we changed the catholyte to that without $CuSO_4$ (catholyte: 0.05 M H_2SO4 + 2.5 M KCl, anolyte: 0.05 M H_2SO_4) and tested the performance of the catalyst for a long time at -200 mA/cm^2. The performance of the catalyst was maintained for a long-time operation. It indicates that the catalyst formed is stable in acidic CO_2R.

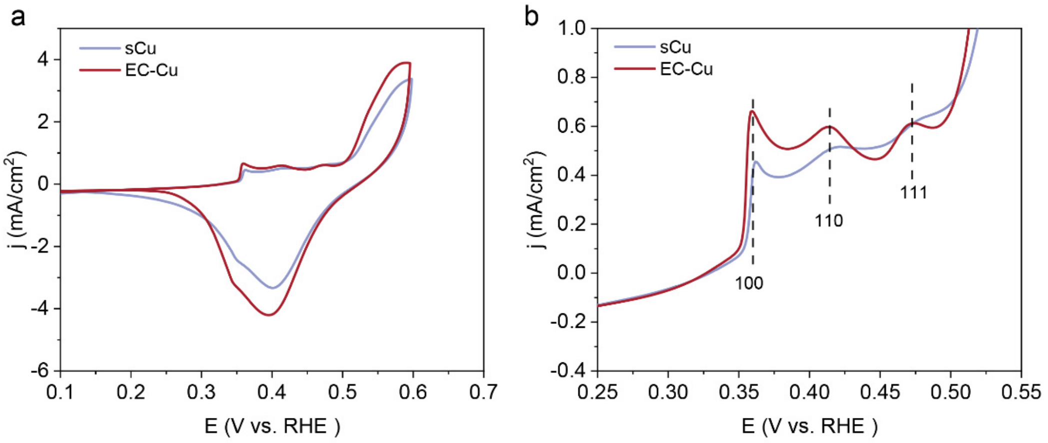

Supplementary Figure 13. Electrochemical adsorption of OH^- on the sCu and EC-Cu ($Cu + OH^- \rightarrow Cu(OH)_{ad} + e^-$). (a) CV curves of sCu and EC-Cu in 1M KOH. (b) Different peaks represent different facets Cu(100), Cu(110), and Cu(111) on the surface of the catalysts. Ratios between different facets on sCu and EC-Cu were quantified by integrating the charges of each peak. The values are 1.2:5.6:1 and 1.3:2.1:1, respectively.

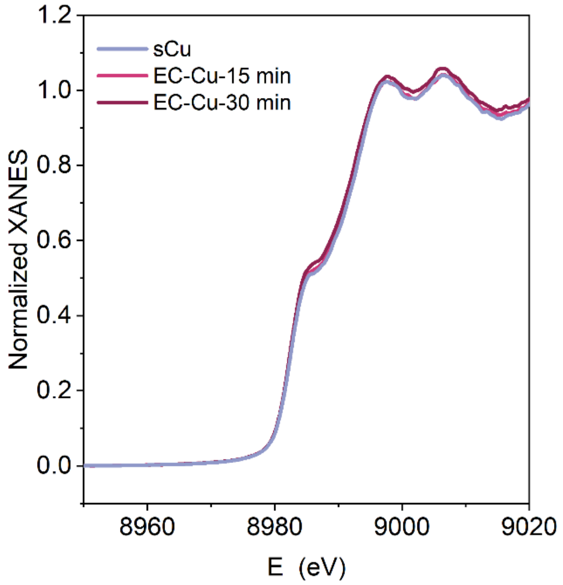

Supplementary Figure 14. Cu-K edge XANES spectra of sCu and EC-Cu with respect to time at 200 mA cm^{-2} in 0.05 M H_2SO4 and 2.5 M KCl containing the copper precursor (3 mM). Before measurement, the sCu was in-situ reduced.

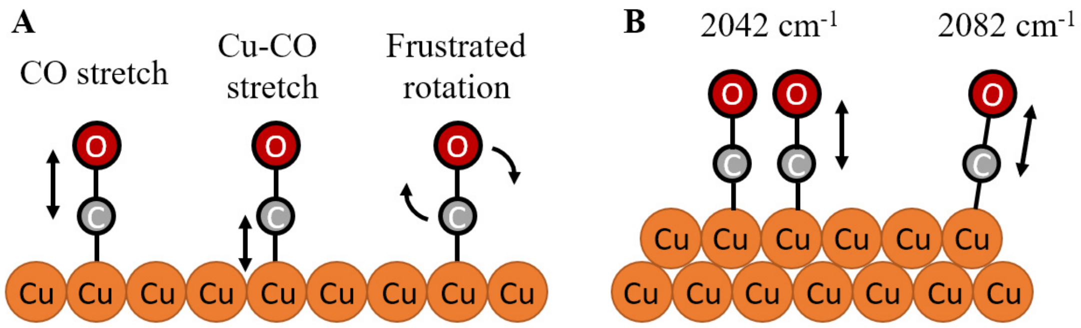

Supplementary Figure S15. Schematics showing (A) the vibrational modes of CO on Cu and (B) the vibrational frequency of different CO_{atop} on Cu.

A schematic of the vibrational modes of CO on Cu is shown in Fig. S21A. Depending on the adsorption site of CO on Cu (e.g., on-top vs. bridge), these modes can have different vibrational frequencies. Based on previous work,[1,2] we assign the Raman peaks from 2000-2100 cm^{-1} to CO stretch vibration (ν(CO)), we assign the peak at 360 and 280 cm^{-1} to Cu-CO stretching (ν(Cu-CO)) and frustrated rotation, respectively. The two atop adsorption configurations of CO on Cu are shown in Fig. S21B. The peak at 2082 cm^{-1} is related to isolated CO on step sites, whereas the peak at 2042 cm^{-1} is related to CO adsorption on terrace sites.

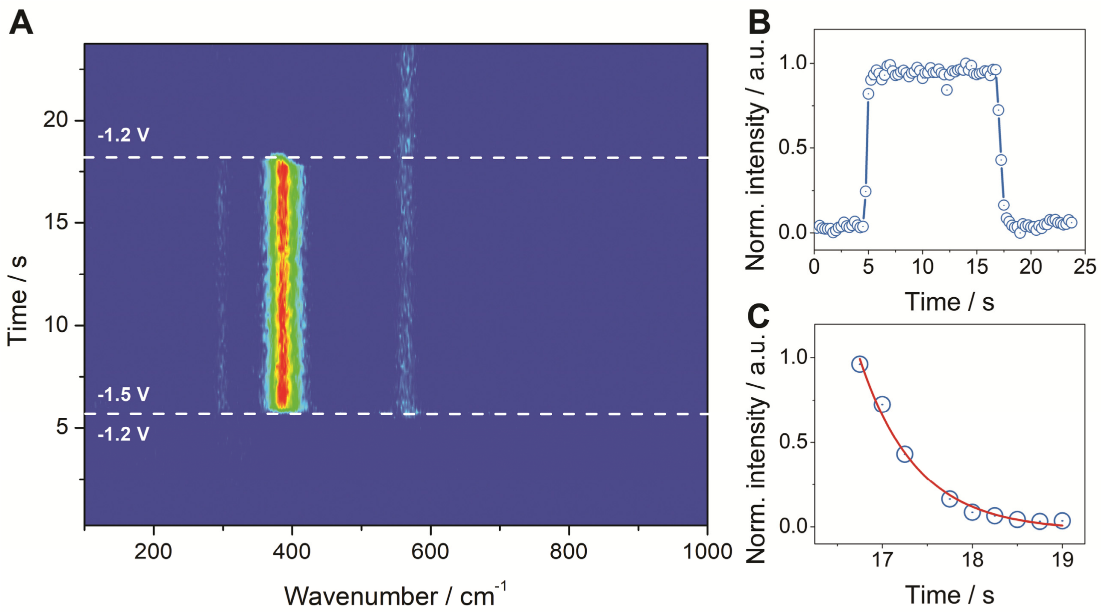

Supplementary Figure 16. Time-resolved *in situ* Raman spectroscopy of EC-Cu prepared on 200 nm of sputtered Cu on PTFE (sCu 200nm). (A) Intensity change of the ν(Cu-CO_{atop}) peak at different potentials. (B) Normalized intensity change of following potential step between -1.2 and -1.5 V. C) Exponential fit to the decay of the ν(CO_{atop}) peak intensity with a time constant of 0.63 s.

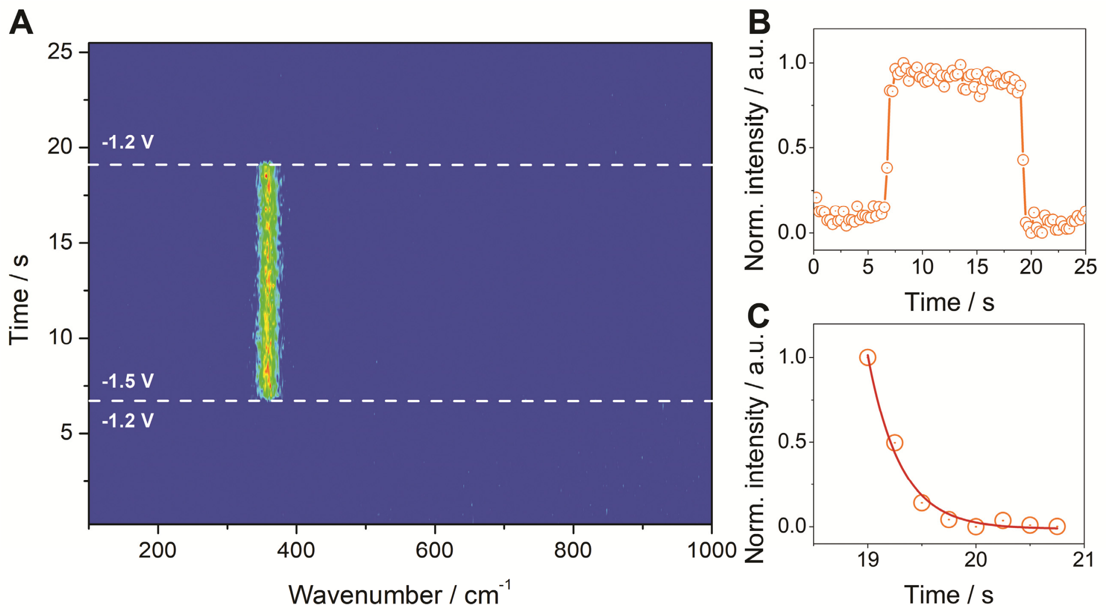

Supplementary Figure 17. Time-resolved *in situ* Raman spectroscopy of sputtered Cu on PTFE (sCu 200nm). (A) Intensity change of the ν(Cu-CO_{atop}) peak at different potentials. (B) Normalized intensity change of following potential step between -1.2 and -1.5 V. C) Exponential fit to the decay of the ν(CO_{atop}) peak intensity with a time constant of 0.30 s.

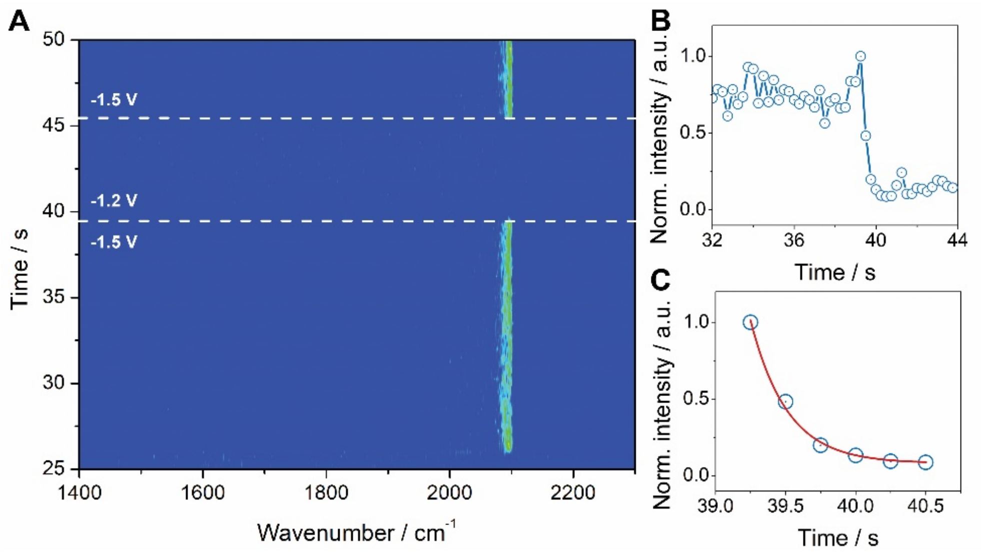

Supplementary Figure 18. Time-resolved *in situ* Raman spectroscopy of EC-Cu prepared on 200 nm of sputtered Cu on PTFE (sCu 200nm) after 7.5 hours of operation. (A) Intensity change of the $\nu(CO_{atop})$ peak at different potentials. (B) Normalized intensity change of following potential step from -1.5 to -1.2 V. (C) Exponential fit to the decay of the $\nu(CO_{atop})$ peak intensity with a time constant of 0.27 s.

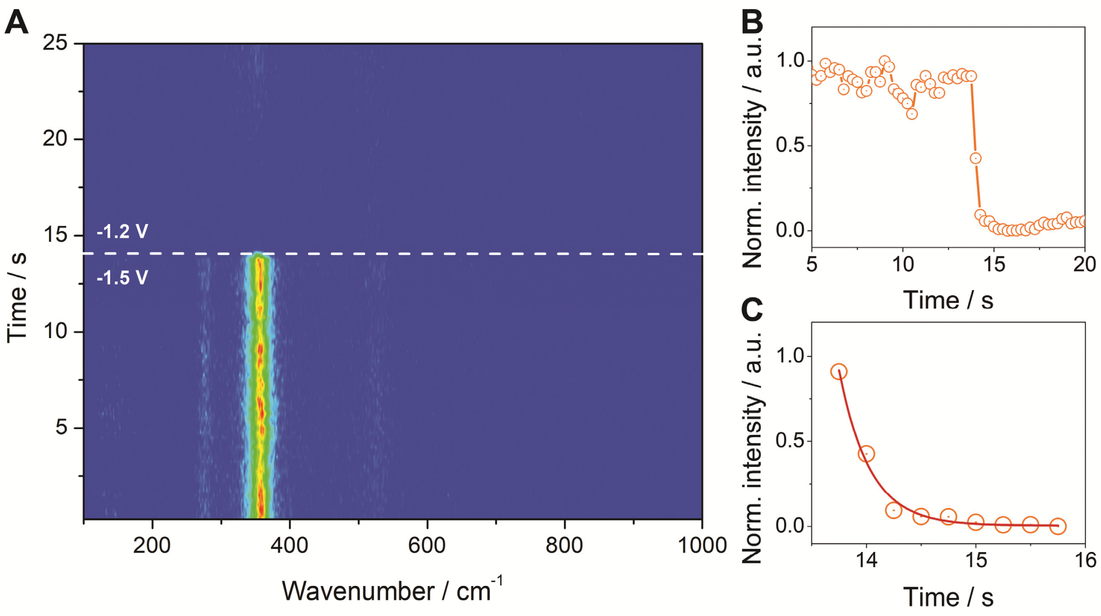

Supplementary Figure 19. Time-resolved *in situ* Raman spectroscopy of EC-Cu prepared on 200 nm of sputtered Cu on PTFE (sCu 200nm) after 7.5 hours of operation. (A) Intensity change of the ν(Cu-CO_{atop}) peak at different potentials. (B) Normalized intensity change of following potential step from -1.5 to -1.2 V. (C) Exponential fit to the decay of the ν(CO_{atop}) peak intensity with a time constant of 0.28 s.

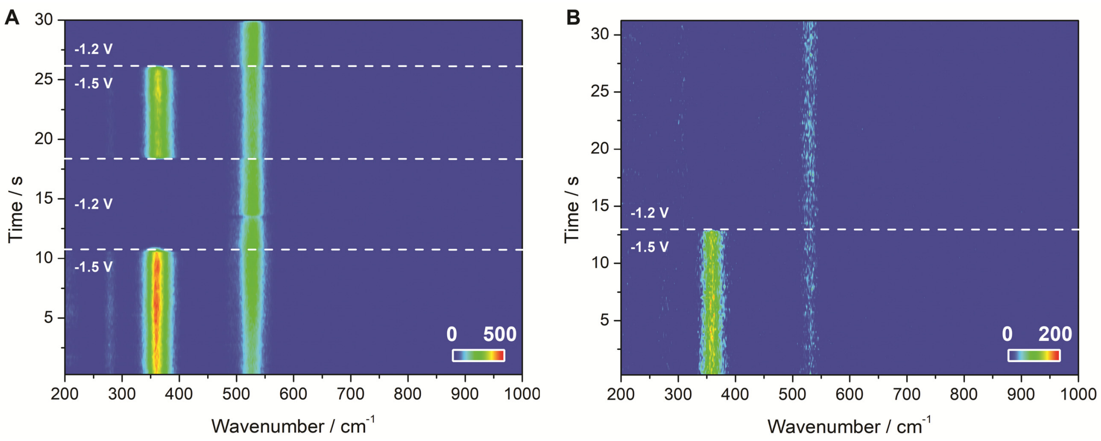

Supplementary Figure S20. Time-resolved in situ Raman spectroscopy of (A) EC-Cu and (B) sCu. Raman spectra were collected at 1-second intervals.

During CO_2R, OH^- ions are generated after H_2O molecules transfer protons to the reaction intermediates on the catalyst surface. Once OH^- ions are generated, they could adsorb on the catalyst surface (OHad) or remain in the solution (OH_{aq}^-) - as the Reviewer has pointed out. Let us first explain why we believe there is OHad on the surface. Upon OH^- chemisorption on the Cu surface, a Cu-OH bond is formed. This metal-oxygen bond – ν(Cu-OH) has a characteristic vibration at around 520-540 cm^{-1}, and we observed a Raman peak at this frequency (Fig. 3, main text). Assigning this peak at 531 cm^{-1} to the ν(Cu-OH) has been proposed in previous publications, and its identity was confirmed using isotopologue experiments.[3-5] One reason this Cu-OH species is observed at negative potentials could be due to hydrogen bonding between the adsorbed hydroxide and the nearby water molecule. While hydroxide ion is considered a weak proton donor, it is an excellent proton acceptor. Thus, the formation of a surface complex [HO_{ad}---HOH] could be expected. Previous DFT calculations and isotopologue experiments have suggested that hydrogen bonding can occur.[3-5] Additionally, this 531 cm-1 peak related to Cu-OH persisted even after reducing the applied potential to -1.2 V – a potential where little or no CO2RR is taking place (lacking COad features in Raman) (Supplementary Fig. S16). If the 531 cm^{-1} peak is related to the hydroxyl group (OHaq−), one would expect this species to either diffuse away from the surface or neutralize with nearby H_3O^+ leading to a quick decline in peak intensity. However, the 531 cm-1 peak persists at -1.2 V for both EC-Cu and sCu samples in Supplementary Fig. S20.

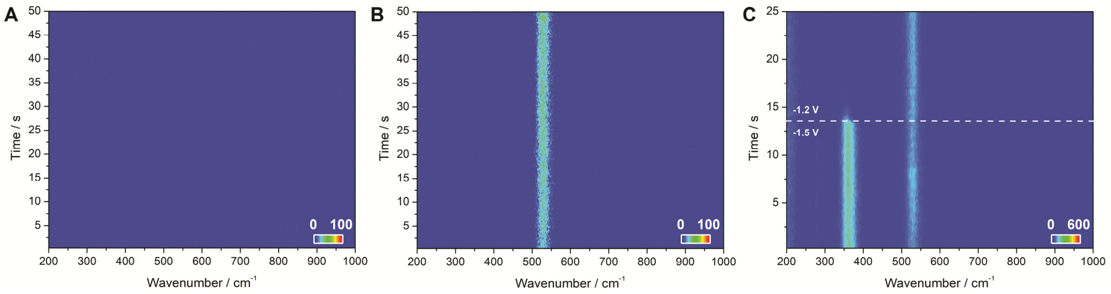

Supplementary Figure S21. Time-resolved in situ Raman spectroscopy of EC-Cu in 0.1 M NaOH. Raman spectra were collected at 1-second intervals. (A) CO_2 free at -0.5 V, (B) CO_2 free at -1.5 V, (C) with CO_2, at -1.2 and -1.5 V.

We now examine (hydrated) OH_{aq}^-. The characteristic vibrational frequencies of OH_{aq}^- is around 3600 cm^{-1},[6] and it has *no* vibrational features at around 531 cm^{-1}. Thus, if all the OH-related species are OH_{aq}^-, one would *not* expect to see the peak at 531 cm^{-1}, which is inconsistent with our Raman results. We also examine the Raman spectrum of EC-Cu in 0.1 M NaOH. If the 531 cm^{-1} peak is related to OH_{aq}^-, then one would expect to see this peak at all potentials. However, the 531 cm^{-1} peak is not observed at -0.5 V (Supplementary Fig. S21A), but it appears when OH_{ad} is formed at a sufficiently negative applied potential (Supplementary Fig. S21B). Since we cannot sufficiently resolve the ν(OH) region in our Raman data, we cannot rule out the existence of OH_{aq}^- and its influence on the CO_2R selectivity.

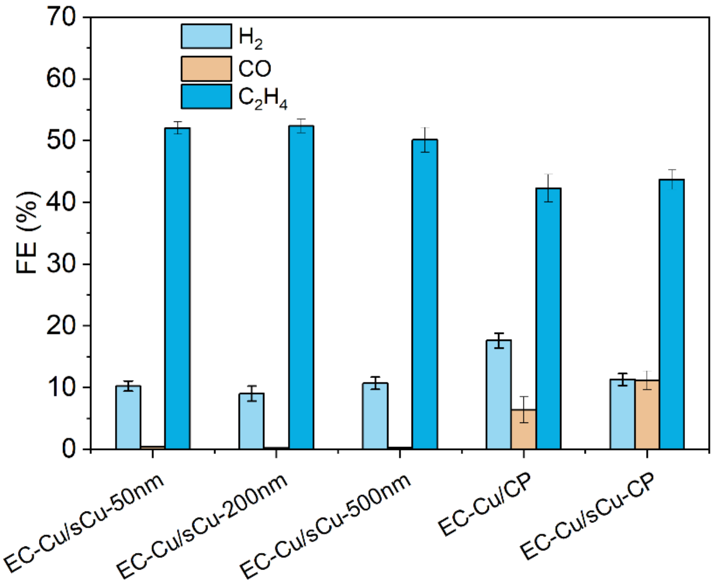

Supplementary Figure 22. *In situ* electrodeposited Cu catalysts on different substrates. We tested the acidic CO_2R performance of EC-Cu on different substrates. sCu-50nm, sCu-200nm, and sCu-500nm are the sputtered Cu on a PTFE GDL with different thicknesses. CP is the carbon paper (Freudenberg H14C9). sCu-CP is the sputtered Cu on the carbon paper (200 nm). The catalysts were deposited at -200 mA/cm^2 for 10 min (0.05 M H_2SO_4, 2.5 M KCl, 7 mM $CuSO_4$, the flow rate of CO_2: 40 sccm). The results indicate that the sputtered Cu on the PTFE, among all the electrodes investigated, is the best substrate for electrodeposition with low HER activity and high C_2H_4 FE. sCu-50nm had similar performance with the sCu-200nm, and the performance of sCu-500nm was a little poorer than that of sCu-50nm and sCu-200nm. The electrodeposited catalysts on carbon paper or sputtered Cu on carbon paper produced a higher CO productivity with lower C_2H_4 and C_{2+} FEs. The error bars correspond to the standard deviation of three independent measurements.

a c b d

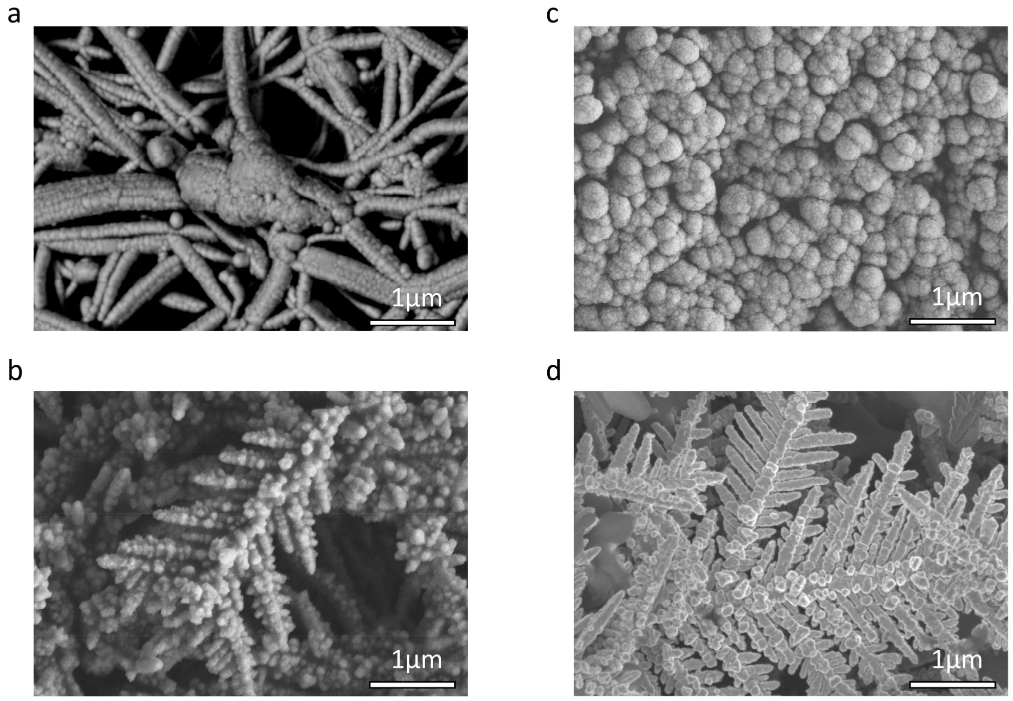

Supplementary Figure 23. SEM images of (a) sCu/PTFE. (b) EC-Cu/PTFE. (c) sCu/carbon paper. (d) EC-Cu/carbon paper.

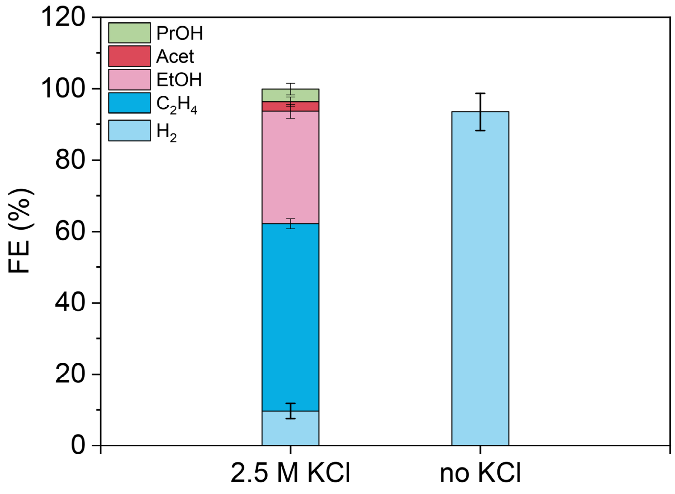

Supplementary Figure 24. Acidic CO_2R product distribution of EC-Cu-2 with (2.5 M) and without KCl in the catholyte. K^+ is necessary to impede hydrogen evolution by suppression of migration of hydronium ions while at the same time promoting CO_2 reduction by stabilization of key intermediates. The error bars correspond to the standard deviation of three independent measurements.

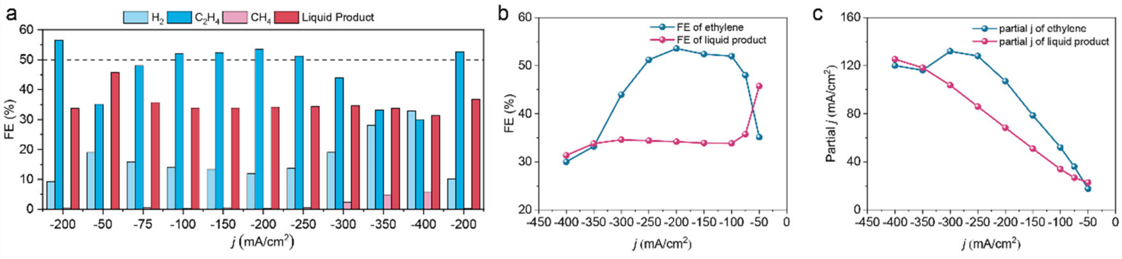

Supplementary Figure 25. Performance of the EC-Cu-2 at different current densities. (a) We deposited the catalyst at -200 mA/cm^2 for 10 min (0.05 M H_2SO_4, 2.5 M KCl, 7 mM $CuSO_4$, the flow rate of CO_2: 40 sccm) and then tested the performance at different current densities from -50 mA/cm^2 to -400 mA/cm^2 (catholyte: 0.05 M H_2SO4 + 2.5 M KCl, anolyte: 0.05 M H_2SO4, CO_2 flow rate: 40 sccm). Finally, we switched back to -200 mA/cm^2. There's an optimal current density window (-75 ~ -250 mA/ cm^2) for good performance in acidic CO_2R. At too low or too high current densities, the performance became bad. If the current densities are too low, the local pH near the catalyst surface will be acidic and the HER activity will dominate. The reason for bad performance at high current densities we guess is the limited CO_2 solubility. When we switched back to -200 mA/cm^2, the good performance came back. Herein, the structure of the catalysts didn't change at high current densities. (b) FE of C_2H_4 and liquid product at different current densities. (c) The partial current density of C_2H_4 and liquid product at different current densities.

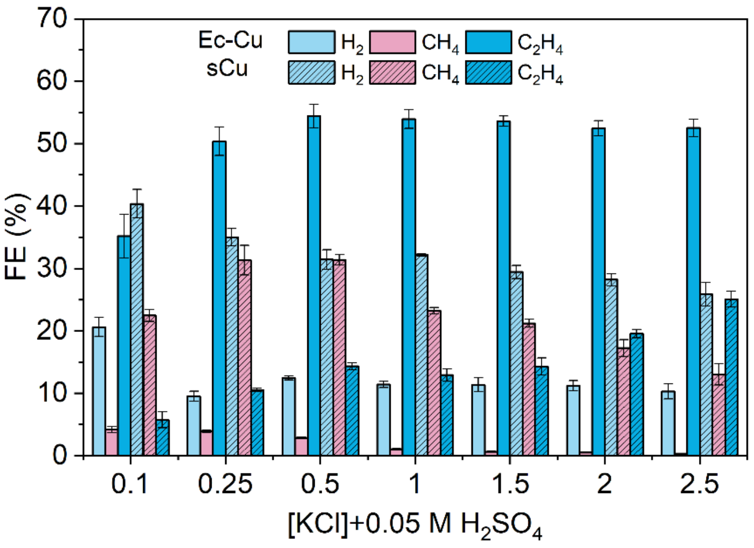

Supplementary Figure 26. Performance of the EC-Cu-2 under different potassium chloride concentrations. The catalysts were deposited at -200 mA/cm^2 for 10 min (0.05 M H_2SO_4, 2.5 M KCl, 7 mM $CuSO_4$, the flow rate of CO_2: 40 sccm) and then tested the performance using different catholyte at -200 mA/cm^2. K^+ is important for locally basic conditions and C-C coupling in the acidic CO_2R. Decreasing the K^+ concentration is predicted to lead to an increase in the HER activity and a reduction of the C-C coupling. The C_2H_4 FE of sCu decreased at lower K^+ concentration, while the EC-Cu produced C_2H_4 and C_{2+} robustly with low HER activity even at 0.25 M K^+. It shows the great advantage of the EC-Cu in C-C coupling and suppressing the HER in acidic CO_2R. The error bars correspond to the standard deviation of three independent measurements.

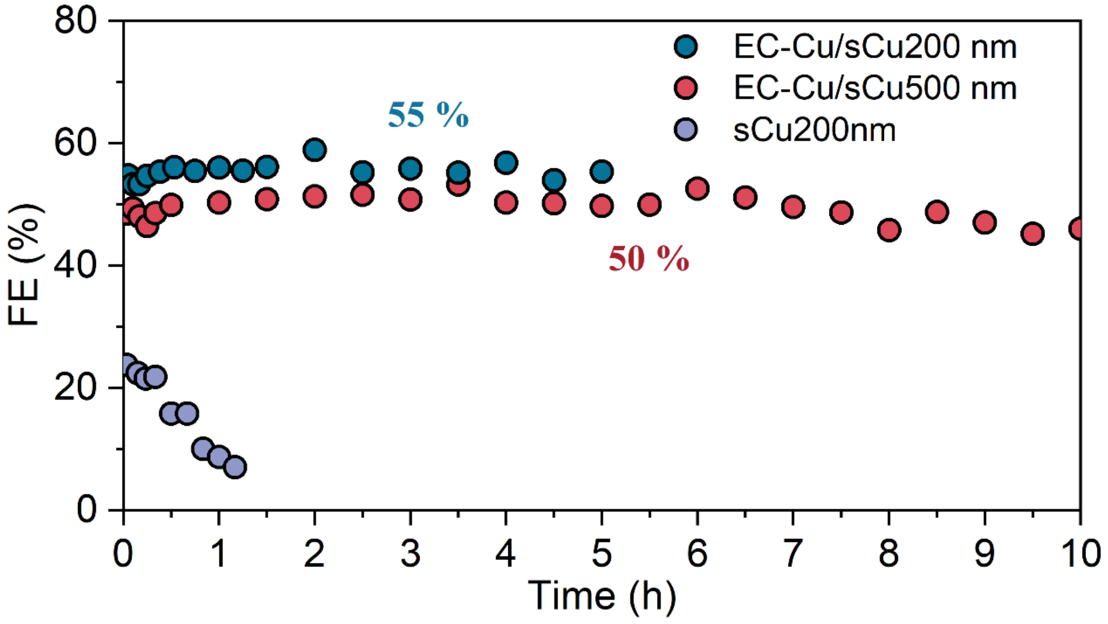

Supplementary Figure 27. Stability of the electrodeposited catalysts and sputtered Cu. We evaluated the stability of EC-Cu and sCu in acidic CO_2R at -200 mA/cm^2 (catholyte: 0.01 M H_2SO4 + 2.5 M KCl, anolyte: 0.05 M H_2SO4, CO_2 flow rate: 40 sccm). The EC-Cu/sCu200nm and EC-Cu/sCu500nm were deposited at -200 mA/cm^2 for 10 min (0.05 M H_2SO_4, 2.5 M KCl, 7 mM $CuSO_4$, the flow rate of CO_2: 40 sccm) on sCu-200 nm and sCu-500 nm. The EC-Cu on thicker sCu had better stability. The stability of EC-Cu in acidic CO_2R was much better than the sCu. We identified two reasons of why the EC-Cu is more stable than the sCu. One reason is the thickness of the catalysts. The thickness of the Cu species on the GDE influences the stability. EC-Cu grows on the sCu and leads to a thicker catalyst layer. The other reason is the much lower H_2 FE of EC-Cu(**Fig.2b-c**). The higher HER activity leads to generation of H_2 bubbles, accelerating the detachment of the catalyst from the GDE.

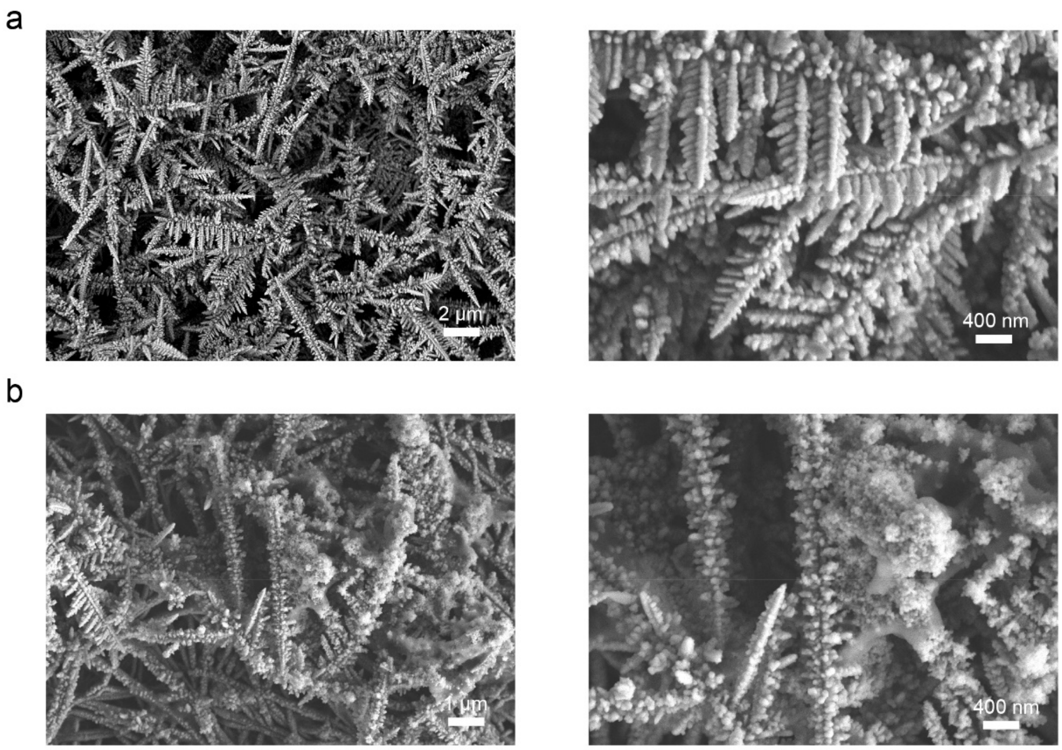

Supplementary Figure 28. SEM illustrated the structure change after a long-time acidic CO_2R operation of the EC-Cu-2. (a) Fresh deposited EC-Cu/sCu200nm (b) EC-Cu/sCu200nm after 7.5 h operation.

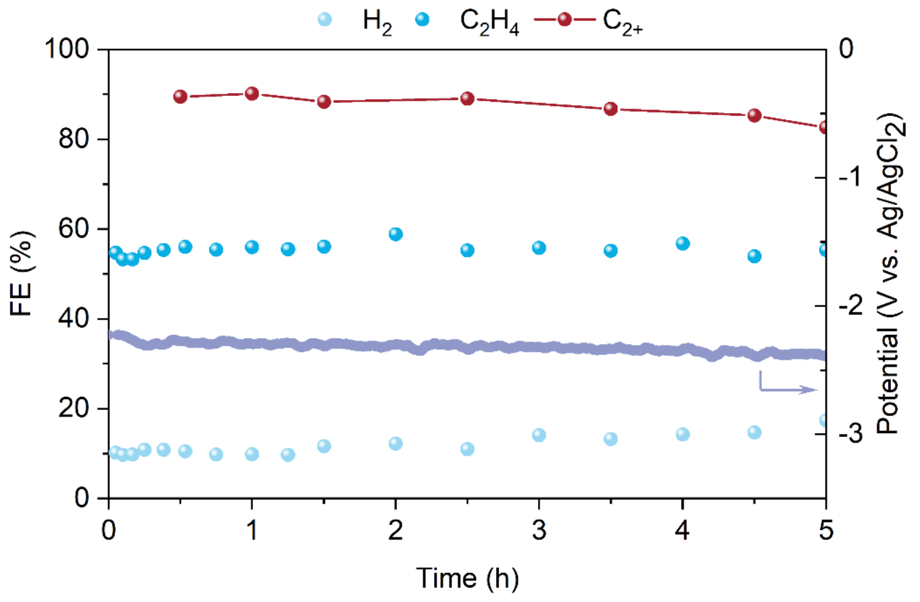

Supplementary Figure 29. Stability of the EC-Cu/sCu200nm. The EC-Cu/sCu200nm was deposited at -200 mA/cm^2 for 10 min (0.05 M H_2SO_4, 2.5 M KCl, 7 mM $CuSO_4$, the flow rate of CO_2: 40 sccm) and then tested the stability at -200 mA/cm^2 (catholyte: 0.01 M H_2SO4 + 2.5 M KCl, anolyte: 0.05 M H_2SO4, CO_2 flow rate: 40 sccm). The FE of H_2, C_2H_4, and C_{2+} were shown.

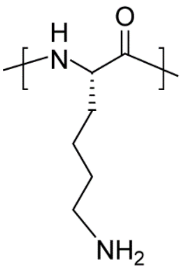

Poly (L-Lys)

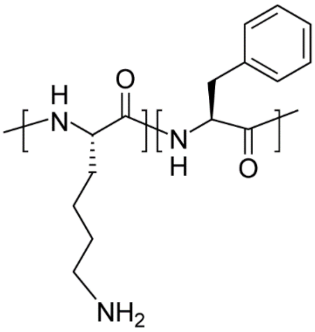

Poly (L-Lys, L-Phe)

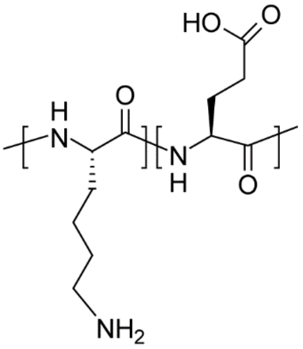

Poly (L-Lys, L-Glu)

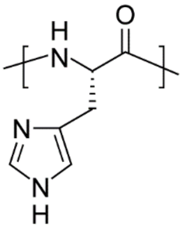

Poly (L-His)

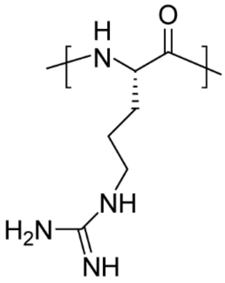

Poly (L-Arg)

Supplementary Figure 30. Structure of the poly(amino acid) we used in this work.

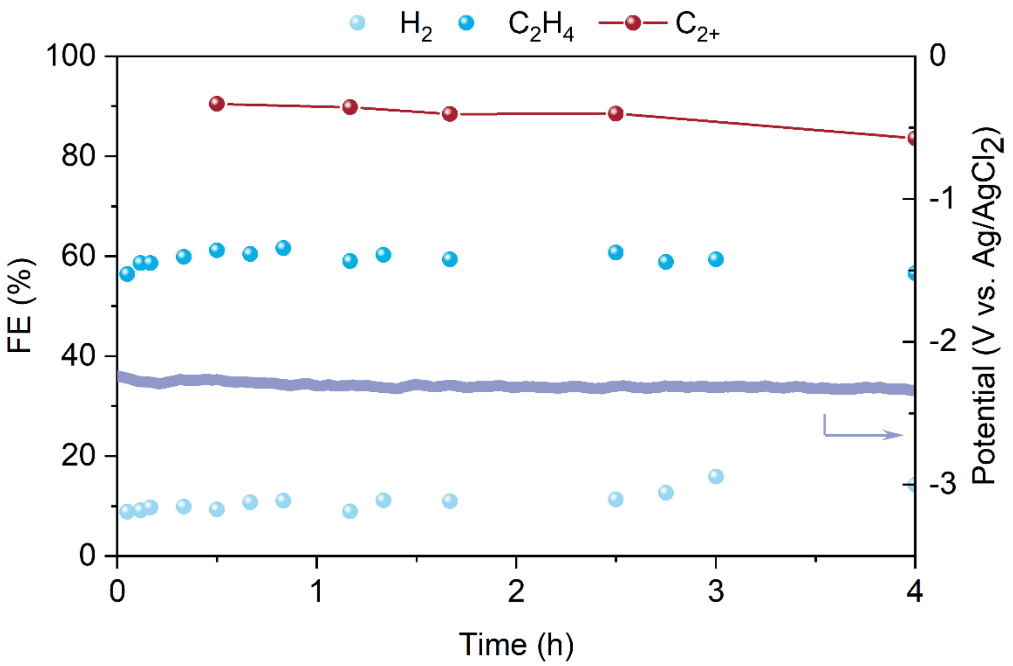

Supplementary Figure 31. Stability of the EC-Cu/sCu200nm with poly(Lys, Phe) additives. The EC-Cu/sCu200nm was deposited at -200 mA/cm^2 for 10 min (0.05 M H_2SO_4, 2.5 M KCl, 7 mM $CuSO_4$, 0.1 mg/ml poly(Lys, Phe), the flow rate of CO_2: 40 sccm) and then tested the stability at -200 mA/cm^2 (catholyte: 0.01 M H_2SO4 + 2.5 M KCl, anolyte: 0.05 M H_2SO4, CO_2 flow rate: 40 sccm). The FE of H_2, C_2H_4, and C_{2+} were shown.

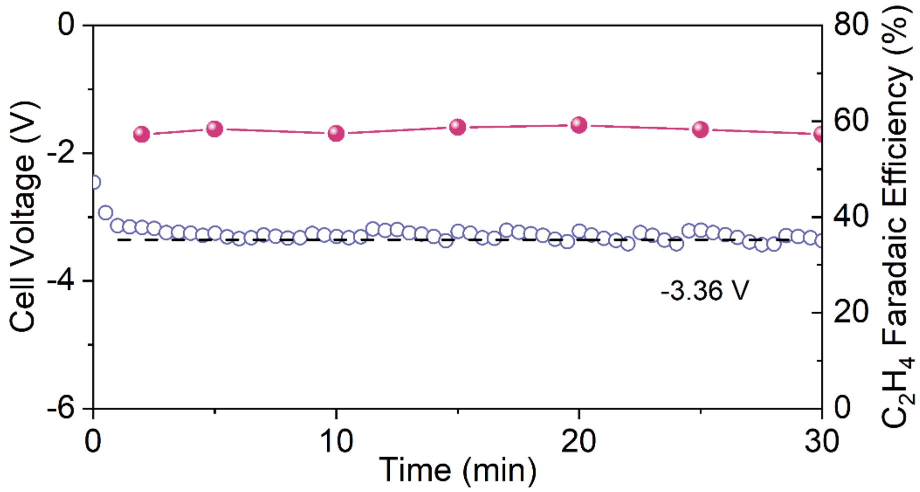

Supplementary Figure 32. Full-cell voltage and FE of the C_2H_4 obtained in a slim flow cell. The EC-Cu/sCu200nm was deposited at -200 mA/cm^2 for 10 min (0.05 M H_2SO_4, 2.5 M KCl, 7 mM $CuSO_4$, 0.1 mg/ml poly(Lys, Phe), and the flow rate of CO_2: 40 sccm) in the flow cell. After electrodeposition, we washed the electrode with DI water and then dried it under a vacuum. The prepared electrode was tested in a slim flow cell (catholyte: 0.01 M H_2SO_4 + 2.5 M KCl, anolyte: 0.05 M H_2SO_4 + 0.6 M K_2SO_4, CO_2 flow rate: 20 sccm).

Supplementary Table 1. Flow rate-dependent energy intensity of producing ethylene

**Parameters**	**10 sccm**	**5 sccm**	**2 sccm**	**1 sccm**	**0.5 sccm**
Cell voltage (V)	3.4	3.4	3.4	3.4	3.4
Faradaic efficiency (%)	61.0	59.3	54.3	50.1	45.0
Current density (mA cm^{-2})	200	200	200	200	200
C_2H_4 Single pass conversion (%)	3.0	5.9	13.5	24.9	44.9
**Electrolyser specific energy distribution (GJ/(tonne ethylene)$^{-1}$)**					
Electrolyser electricity	230.5	237.1	258.9	280.6	312.4
Cathode separation	54.6	29.1	14.8	10.2	8.5
Anode separation (Carbonate)	0.0	0.0	0.0	0.0	0.0
Carbonate regeneration	0.0	0.0	0.0	0.0	0.0
Overall energy	285.1	266.2	273.7	290.8	320.9

Supplementary Table 2. High energy efficiency conditions (50 sccm for the Science reference and 10 sccm for this work)

Parameters	Current best (Science 2021[7])	This work
Cell voltage (V)	4.2	3.4
Faradaic efficiency (%)	28	61
Current density (mA cm^{-2})	1200	200
C_2H_4 Single pass conversion (%)	2.1	3.0
**Electrolyser specific energy distribution (GJ/(tonne ethylene)$^{-1}$)**		
Electrolyser electricity	620.3	230.5
Cathode separation	86.2	54.6
Anode separation (Carbonate)	0.0	0.0
Carbonate regeneration	0.0	0.0
**Overall energy**	**706.5**	**285.1**

Supplementary Table 3. High single-pass conversion efficiency conditions (3 sccm for the Science reference and 0.5 sccm for this work)

Parameters	Current best (Science 2021[7])	This work
Cell voltage (V)	4.2	3.4
Faradaic efficiency (%)	26	45.0
Current density (mA cm^{-2})	1200	200
C_2H_4 Single pass conversion (%)	26.4	44.9
**Electrolyser specific energy distribution (GJ/(tonne ethylene)$^{-1}$)**		
Electrolyser electricity	620.3	312.4
Cathode separation	28.8	8.5
Anode separation (Carbonate)	0.0	0.0
Carbonate regeneration	0.0	0.0
**Overall energy**	**649.1**	**320.9**

Supplementary Table 4. Operation parameter λ at different CO_2 flow rates.

**Flow rate (sccm)**	**40**	**20**	**10**	**5**	**2**	**1**	**0.5**
λ of C_2H_4	265.2	136.2	65.8	33.8	14.8	8.0	4.5
λ of C_1	50931.1	35651.8	15728.7	5570.6	1407.3	74.3	51.4
λ of C_{2+}	175.0	89.0	45.1	22.7	9.8	5.4	2.9

Here, we defined the operation parameter λ as the ratio between the mole rate of supplied CO_2 and the formation rate of C_2H_4, C_1, and C_{2+} by CO_2R[8,9].

Supplementary Note 1

The ν(CO_{atop}) peak position can be influenced by many factors. With increasing CO coverage, dipolar coupling[10], and chemical shifts[11] are effects that can blue shifts and red shifts the ν(CO_{atop}), respectively. With the application of electrode potentials, the back-donation from Cu to CO (d-2π*) is affected—this results in the well-known Stark effect.[3] When comparing our *in situ* Raman results of sCu and EC-Cu in Figure 3, Stark shift is less relevant since the same potential is applied to both samples. Dipole-dipole interaction between CO molecules has been shown to have little influence on the ν(CO_{atop}) frequency according to Iijima *et al.* As for the chemical shift, saturation level of θ_{CO} has been reached at -1.5 V for both sCu and EC-Cu, thus, a chemical effect would have red-shifted the ν(CO_{atop}) by a similar amount. Summing these arguments, we believe the significant red shift in the ν(CO_{atop}) frequency experience by the EC-Cu sample is a result of interactions with co-adsorbed OH at the surface.

In the time-resolved Raman studies, we chose to investigate the surface CO coverage (θ_{CO}) at -1.2 V and -1.5V—these potential values correspond to low (<1 $mAcm^{-2}$) and high (>15 $mAcm^{-2}$) rate conditions. The electroattractive interactions between co-adsorbed CO and OH can result in longer C–O bond and a red-shift of the ν(CO_{atop}) peak[12]. DFT calculations have shown that the wavenumber of CO stretching in the presence of one OH is 2020 cm^{-1} and in the absence of OH is in 2070 cm^{-1}. More extensive interaction of two OH and two CO molecules have led to a peak at 1951 cm^{-1}, which is similar to our observation of a broad peak at 1972 cm^{-1}—a feature absent for sCu.

Supplementary Note 2

Example energy intensity calculation for acidic media CO_2-to-ethylene conversion. This section presents a sample energy intensity calculation for the CO_2-to-ethylene conversion in a slim flow with acidic electrolytes. The model used the experimentally achieved Faradaic efficiency, full-cell potential, single pass conversion efficiency, and current density as the input to calculate the energy intensity associated with the electrolyser electricity and cathode separation (more specifically, under the operation mode that enables the lowest energy intensity in CO_2-to-ethylene conversion). These metrics include an ethylene Faradaic efficiency of 59.3%, a full-cell voltage of 3.4 V, a CO_2-to-ethylene single pass conversion efficiency of 5.9%, and a current density of 200 mA cm^{-2} (the performance metrics achieved with the acidic CO_2R electrolyser under the CO_2 flow rate of 5 sccm, Supplementary Table 1-3).

Electrolyser electricity. We first calculate the production rate of ethylene on a molar basis (mol/s) for a target ethylene production capacity of 100 tons.

$$Production\ rate \left[\frac{mol}{s}\right] = \frac{Production\left[\frac{g}{day}\right]}{molecular\ weight_{ethylene}\left[\frac{g}{mol}\right] \times 86400\left[\frac{s}{day}\right]} \quad (1a)$$

$$Production\ rate\ \left[\frac{mol}{s}\right] = \frac{100 \times \frac{10^6 g}{day}}{\frac{28\ g}{mol} \times \frac{86400s}{day}} = 41.335\frac{mol}{s} \tag{1b}$$

Then, we calculate the current needed to achieve ethylene production at this rate for an experimentally achieved ethylene FE of 59.3%:

$$Total\ current\ required\ [A] = \frac{production\ rate\ \left[\frac{mol}{s}\right] \times electrons\ transferred \times Faraday's\ Constant}{FE_{ethylene}[decimal]} \tag{2a}$$

$$Total\ current\ needed\ [A] = \frac{41.335\frac{mol}{s} \times 12 \times 96485\frac{sA}{mol}}{0.593} = 80\ 705\ 716\ A \tag{2b}$$

Then, the total current is multiplied by the full-cell potential (3.4 V) to determine the power required:

$$Power\ \text{required}\ [W] = Total\ current\ needed\ [A] \times Cell\ voltage\ [V] = 80\ 705\ 716\ A \times 3.4\ V = 274\ 399\ kW \tag{3}$$

Then, the energy needed to run the plant for one day (to achieve the production capacity of 100 tons) is calculated multiplying by 24 hours:

$$Electricity\ energy\ required\ \left[\frac{GJ}{ton^{-1}}\right] = \frac{Power\ required\ [W] \times 24[\text{h}]}{daily\ production\ [ton]} = \frac{274\ 399\ \text{k}W \times 24\ \text{h} \times 0.0036\frac{\text{GJ}}{\text{kWh}}}{100\ ton} = \mathbf{237.1}\ \frac{\boldsymbol{GJ}}{\boldsymbol{ton}} \tag{4}$$

Cathode separation. In order to recover the product ethylene from the cathodic gas stream, a pressure swing adsorption (PSA) is applied at the downstream of the acidic CO_2R electrolyser. The model considers the energy input associated with the capital and operating costs of the PSA separation unit based on a model for biogas upgrading. The cathodic gas stream is modeled to account for the presence of ethylene, unreacted CO_2 and side-reaction product hydrogen. The model uses a scaling factor of 0.7 and energy requirement of 0.25 kWh m^{-3} for a cost of \$1 989 043 (for a flow rate of 1000 m^3 h^{-1}). With these, the operating energy input for the PSA module is calculated as follows.

$$PSA\ operating\ energy\ \left[\frac{\text{kWh}}{ton\ ethylene}\right] = 0.25\ \frac{kWh}{m^3} \times flow\ rate \left[\frac{m^3}{h}\right] \times 24\frac{h}{day} \qquad (5a)$$

Before this, we first determine the flow rate at the cathodic gas stream: flow rate of ethylene under the ideal conditions is calculated as follows.

$$Output\ ethylene\ flow\ rate \left[\frac{m^3}{h}\right] = \frac{100 \times 10^6\ g \times 8.314\, Jmol^{-1}K^{-1} \times 298K}{28\frac{g}{mol} \times 101\ 300\ Pa \times 24\frac{h}{day}} = 3639\ \frac{m^3}{h} \qquad (6)$$

The model also considers the flow rates of product ethylene, unreacted CO_2, and side-product hydrogen at the cathodic gas stream. The unreacted CO_2's flow rate is calculated using the single conversion efficiency towards ethylene (by assuming a constant pressure). Herein, we note that the single pass conversion efficiency is a function of the amount of CO_2 being reduced and that of the CO_2 amount being supplied to the cathodic stream. For a single pass CO_2-to-ethylene conversion efficiency of 5.9%, we can determine the output CO_2 flow rate as follows.

$$OutputCO_2\ flow\ rate \left[\frac{m^3}{h}\right] =$$

$$ethylene\ flow\ rate \left[\frac{m^3}{h}\right] \times molar\ ratio\ \left[\frac{CO_2}{ethylene}\right] \times \left(\frac{100 - single\ pass\ conversion[\%]}{single\ pass\ conersion[\%]}\right) \quad (7a)$$

$$Output\ CO_2\ flow\ rate \left[\frac{m^3}{h}\right] = 3639\frac{m^3}{h} \times 2 \times \frac{100 - 5.9}{5.9} = 116077\ \frac{m^3}{h} \qquad (7b)$$

We can then determine the current toward hydrogen as follows.

$$Current\ toward\ \text{hydrogen}\ [A] = Total\ current\ \text{required}\ [A] \times \frac{100 - FE_{ethylene}[\%]}{100} \qquad (8a)$$

$$Current\ toward\ \text{hydrogen}\ [A] = 80\ 705\ 716\ A \times \frac{100 - 59.3}{100} = 32\ 8472\ 26\ A \qquad (8b)$$

The hydrogen's production rate can be described as follows.

$$Hydrogen\ production\ \left[\frac{mol}{h}\right] = \frac{Current\ toward\ \text{hydrogen}\ [A] \times 3600\frac{s}{h}}{2\frac{electrons}{Hydrogen\ product} \times Faraday's\ Constant} \qquad (9a)$$

$$Hydrogen\ production\ \left[\frac{mol}{h}\right] = \frac{32\ 847\ 226\ A \times 3600\frac{s}{hour}}{2\frac{electrons}{Hydrogen\ product} \times 96485\frac{sA}{mol}} = 612\ 789\ \frac{mol}{h} \qquad (9b)$$

Assuming an ideal gas under standard conditions, we calculate the hydrogen's flow rate:

$$flow\ rate_{Hydrogen}\left[\frac{m^3}{h}\right] = \frac{Hydrogen\ production\left[\frac{mol}{h}\right] \times 8.314\frac{J}{mol \times K} \times 298K}{101.3 \times 10^3 Pa} \quad (10a)$$

$$flow\ rate_{Hydrogen}\left[\frac{m^3}{h}\right] = \frac{612\ 789\frac{mol}{h} \times 8.314\frac{J}{mol \times K} \times 298K}{101.3 \times 10^3 Pa} = 14\ 987\ \frac{m^3}{h} \quad (10b)$$

We can then calculate the total flow rate at the cathodic downstream by summing the flow rates of ethylene, unreacted CO_2, and hydrogen.

$$flow\ rate\left[\frac{m^3}{h}\right] = (3639 + 116077 + 14987)\frac{m^3}{h} = 134\ 703\frac{m^3}{h} \quad (11)$$

With the final output flow rate, we can calculate the energy required per ton of ethylene produced as follows:

$$PSA\ Energy\left[\frac{\text{GJ}}{\text{ton ethylene}}\right] = 0.25\ \frac{\text{kWh}}{\text{m}^3} \times 134\ 703\frac{\text{m}^3}{\text{h}} \times 24\frac{\text{h}}{\text{day}} \times \frac{0.0036\ \text{GJ kWh}^{-1}}{100\ \text{ton ethylene}}$$
$$= \mathbf{29.1}\ \frac{\textbf{GJ}}{\textbf{ton ethylene}} \quad (5b)$$

Supplementary Reference

1. An, H. Y. *et al.* Sub-Second Time-Resolved Surface-Enhanced Raman Spectroscopy Reveals Dynamic CO Intermediates during Electrochemical CO2 Reduction on Copper. *Angew Chem Int Edit* **60**, 16576-16584 (2021).
2. Zhan, C. *et al.* Revealing the CO Coverage-Driven C-C Coupling Mechanism for Electrochemical CO2 Reduction on Cu2O Nanocubes via Operando Raman Spectroscopy. *Acs Catalysis* **11**, 7694-7701 (2021).
3. Niaura, G. Surface-enhanced Raman spectroscopic observation of two kinds of adsorbed OH- ions at copper electrode. *Electrochim Acta* **45**, 3507-3519 (2000).
4. Moradzaman, M. & Mul, G. In Situ Raman Study of Potential-Dependent Surface Adsorbed Carbonate, CO, OH, and C Species on Cu Electrodes During Electrochemical Reduction of CO2. *Chemelectrochem* **8**, 1478-1485 (2021).
5. Bodappa, N. *et al.* Early Stages of Electrochemical Oxidation of Cu(111) and Polycrystalline Cu Surfaces Revealed by in Situ Raman Spectroscopy. *Journal of the American Chemical Society* **141**, 12192-12196 (2019).
6. Itoh, T., Kajita, T., Maeda, T. & Kasuya, A. In Situ Surface-enhanced Raman Analysis of Water Libration on Silver Electrode in Various Alkali Hydroxide Aqueous Solutions. *Electrochemistry* **82**, 396-400 (2014).
7. Huang, J. E. *et al.* CO2 electrolysis to multicarbon products in strong acid. *Science* **372**, 1074-+ (2021).
8. Krause, R. *et al.* Industrial Application Aspects of the Electrochemical Reduction of CO2 to CO in Aqueous Electrolyte. *Chem. Ing. Tech.* **92**, 53-61 (2020).
9. Haas, T., Krause, R., Weber, R., Demler, M. & Schmid, G. Technical photosynthesis involving CO2 electrolysis and fermentation. *Nat. Catal.* **1**, 32-39 (2018).
10. Woodruff, D. P., Hayden, B. E., Prince, K. & Bradshaw, A. M. Dipole coupling and chemical shifts in IRAS of CO adsorbed on Cu(110). *Surf. Sci.* **123**, 397-412 (1982).
11. Hayden, B. E., Kretzschmar, K. & Bradshaw, A. M. An infrared spectroscopic study of CO on Cu(111): The linear, bridging and physisorbed species. *Surf. Sci.* **155**, 553-566 (1985).
12. Gunathunge, C. M., Li, J., Li, X., Hong, J. J. & Waegele, M. M. Revealing the Predominant Surface Facets of Rough Cu Electrodes under Electrochemical Conditions. *ACS Catal.* **10**, 6908-6923 (2020).
